# Fine-Scale Genetic Structure and Demographic History in the Miyako Islands of the Ryukyu Archipelago

**DOI:** 10.1093/molbev/msab005

**Published:** 2021-01-12

**Authors:** Masatoshi Matsunami, Kae Koganebuchi, Minako Imamura, Hajime Ishida, Ryosuke Kimura, Shiro Maeda

**Affiliations:** 1 Department of Advanced Genomic and Laboratory Medicine, Graduate School of Medicine, University of the Ryukyus, Nishihara-Cho, Japan; 2 Advanced Medical Research Center, Faculty of Medicine, University of the Ryukyus, Nishihara-Cho, Japan; 3 Division of Clinical Laboratory and Blood Transfusion, University of the Ryukyus Hospital, Nishihara-Cho, Japan; 4 Department of Human Biology and Anatomy, Graduate School of Medicine, University of the Ryukyus, Nishihara-Cho, Japan

**Keywords:** Ryukyu archipelago, biobank, Japanese, insular biogeography, population structure, demographic history

## Abstract

The Ryukyu Archipelago is located in the southwest of the Japanese islands and is composed of dozens of islands, grouped into the Miyako Islands, Yaeyama Islands, and Okinawa Islands. Based on the results of principal component analysis on genome-wide single-nucleotide polymorphisms, genetic differentiation was observed among the island groups of the Ryukyu Archipelago. However, a detailed population structure analysis of the Ryukyu Archipelago has not yet been completed. We obtained genomic DNA samples from 1,240 individuals living in the Miyako Islands, and we genotyped 665,326 single-nucleotide polymorphisms to infer population history within the Miyako Islands, including Miyakojima, Irabu, and Ikema islands. The haplotype-based analysis showed that populations in the Miyako Islands were divided into three subpopulations located on Miyakojima northeast, Miyakojima southwest, and Irabu/Ikema. The results of haplotype sharing and the *D* statistics analyses showed that the Irabu/Ikema subpopulation received gene flows different from those of the Miyakojima subpopulations, which may be related with the historically attested immigration during the Gusuku period (900 − 500 BP). A coalescent-based demographic inference suggests that the Irabu/Ikema population firstly split away from the ancestral Ryukyu population about 41 generations ago, followed by a split of the Miyako southwest population from the ancestral Ryukyu population (about 16 generations ago), and the differentiation of the ancestral Ryukyu population into two populations (Miyako northeast and Okinawajima populations) about seven generations ago. Such genetic information is useful for explaining the population history of modern Miyako people and must be taken into account when performing disease association studies.

## Introduction

**Fig. 1.**
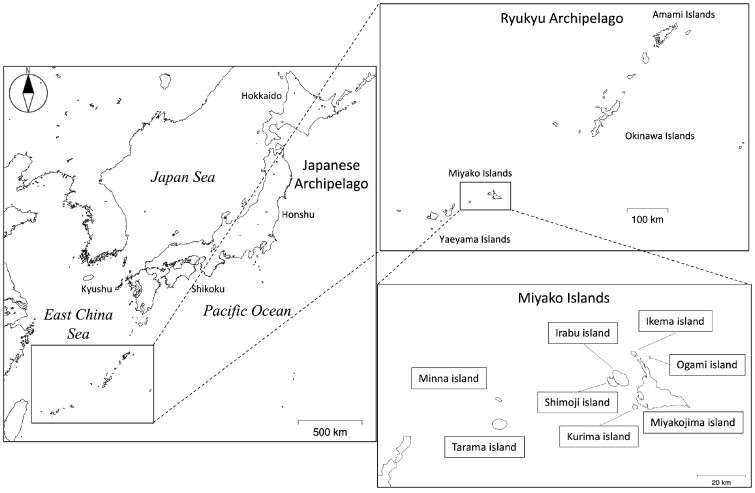
Geographic location of the Ryukyu Archipelago. The Japanese Archipelago includes Hokkaido, Honshu, Shikoku, and Kyushu islands. The Ryukyu Archipelago includes Amami, Okinawa, Miyako, and Yaeyama Islands. The Miyako Islands comprise eight islands: Ikema, Irabu, Kurima, Minna, Miyakojima, Ogami, Shimoji, and Tarama.

The Ryukyu Archipelago makes up the southernmost islands of Japan (fig. 1) and mainly consists of the Amami, Okinawa, Miyako, and Yaeyama Islands. The Miyako Islands are located between the Yaeyama and Okinawa Islands and are comprised of eight islands: Ikema, Irabu, Kurima, Minna, Miyakojima, Ogami, Shimoji, and Tarama (here, “-jima” is used for “Island” to distinguish it from “Islands”). In these islands, about 55,000 individuals are living within a total area of 204 km^2^ (Miyakojima City Board of Education 2012). The Ryukyu Archipelago is surrounded by the Pacific Ocean and the East China Sea. Therefore, the people on these islands are considered to have been isolated from other populations, and individual island groups were also isolated.

Archaeological studies have shown that the Miyako Islands have a unique history (supplementary table 1, Supplementary Material online). On Miyakojima, human bones dating to 31–28 kilo before present (BP) were excavated from the Pinza-Abu Cave site (Sakura 1985), indicating that human settlement on the island dates back to the Paleolithic period. In the Neolithic period when the Jomon culture reached the Okinawa Islands, however, there was a long archaeological blank in the record on the Miyako Islands. About 4,200–3,500 years ago, the Shimotabaru culture emerged mainly in the Yaeyama Islands and on Tarama in the Miyako Islands (Asato 1993). Then, the Aceramic culture (2,500–900 BP), which is characterized by giant clam shell adzes, appeared on the Miyako and Yaeyama Islands (Asato 1993). These cultures are recognized as being distinct from the Jomon culture on the Japanese mainland. The origin of the people who formed the Shimotabaru and Aceramic cultures remains unknown. Immigration waves from the Okinawa Islands to the Miyako and Yaeyama Islands during the Gusuku period (900 − 500 BP) may contribute to forming the current population structure of the Ryukyu Archipelago (Miyakojima City Board of Education 2012). However, the cultures and languages among the present island groups in the Ryukyu Archipelago and among the regions in the Miyako Islands are very diverse, as demonstrated by ethnological and linguistic studies (Pellard 2015).

Genome-wide single-nucleotide polymorphisms (SNPs) data have provided a clear picture of the current and previous population structure in Japan. Within Japan, there are two different clusters, the Hondo cluster on the Japanese Archipelago and the Ryukyu cluster on the Ryukyu Archipelago (Yamaguchi-Kabata et al. 2008). By narrow definition, the Japanese Archipelago does not include the Ryukyu Archipelago; therefore, we hereafter consider the Japanese Archipelago and the Ryukyu Archipelago as independent archipelagoes in this study. Phylogenetic analysis using genome-wide SNPs has also shown that the Ryukyu people show greater sharing of genetic components with the Ainu people than the Hondo people do (Jinam et al. 2012). Among the island groups of the Ryukyu Archipelago, there is genetic differentiation, especially between groups in the Okinawa and Miyako Islands, and there is little genetic affinity between aboriginal Taiwanese and any of the Ryukyu peoples (Sato et al. 2014). In addition, it has been suggested that the Paleolithic people in the Ryukyu Archipelago are not the main ancestors of modern Ryukyu people, but rather that the modern Ryukyu people are descendants of migrations from the Japanese Archipelago in the Neolithic Period or later (Sato et al. 2014). These observations suggest that people living in the Miyako Islands have a unique genetic background, which is different from that of people on other Ryukyu islands, and these differences may contribute to the establishment of their original culture. However, a detailed population structure of each island group within the Ryukyu Archipelago has not yet been elucidated.

Understanding the regional population structure is also important for medical and human genetic research. Genome-wide association studies (GWAS) have identified many genetic loci associated with disease traits (Tam et al. 2019), but, on the other hand, insufficient sample size and/or the presence of population stratification due to the inclusion of genetically diverse populations may skew the results of GWAS (Voight and Pritchard 2005). The recent development of haplotype-based methods for population genetics is more sensitive for detecting differences among relatively close populations (Leslie et al. 2015; Novembre and Peter 2016; Kerminen et al. 2017; Takeuchi et al. 2017; Byrne et al. 2018; Gilbert et al. 2019). Therefore, information obtained from these methods is expected to be useful for identifying additional loci associated with disease traits (Locke et al. 2019).

Insular regions are different from continents with respect to human migration and show a characteristic population structure. Because human migrations to islands are geographically restricted, most island populations are small and historically isolated. These populations might experience strong genetic bottlenecks and show relatively unique and uniform genetic backgrounds which differ from those of other populations. GWAS has been shown to be useful for identifying novel variants associated with disease traits in isolated populations, since unique variants with large effect might be conserved in these isolated populations under specific circumstances (Moltke et al. 2014; Lettre and Hirschhorn 2015).

In this study, we examined the genotypes of people in the Miyako Islands using genome-wide SNPs arrays as a part of the Okinawa Bioinformation Bank (OBi) Project, and we revealed that the population in the Miyako Islands was divided into three subpopulations.

## Results

### Fine-Scale Genetic Structure within the Miyako Islands

**Fig. 2.**
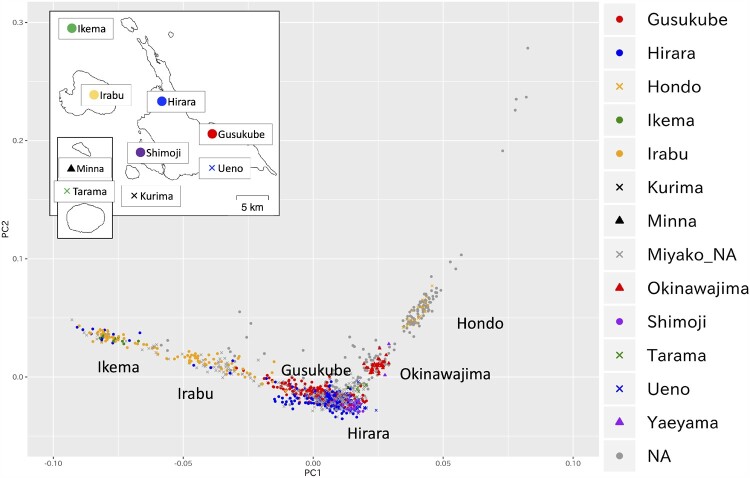
PCA plot using genotype data for 491,109 SNPs from 1,098 individuals. We calculated PC values using samples collected in the Miyako Islands. Based on the birthplace information for grandparents of participants, we colored individuals having four grandparents from the same region among Miyako Islands, Okinawajima island, Hondo, and Yaeyama Islands. Gray circles labeled as NA denote individuals having at least one of four grandparents with a different birthplace from the others. Detailed localities in Miyako Islands are shown in upper left inset. Gray cross marks labeled as Miyako_NA denote individuals having four grandparents from Miyako Islands, but detailed address is unknown or different.

**Fig. 3.**
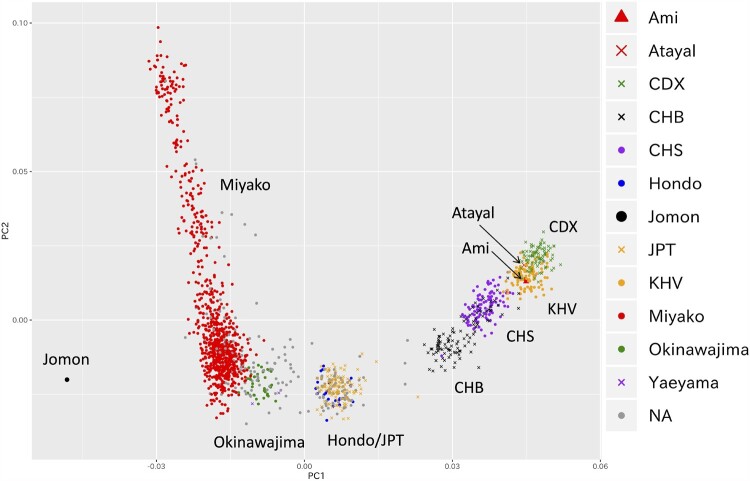
A PCA plot using genotype data for Miyako samples and publicly available East Asian genotype data. A PCA plot for East Asians from the 1000 genomes project, aboriginal Taiwanese (Ami and Atayal), ancient Japanese (Jomon), and participants in this study, Hondo, Miyako, Okinawajima, and Yaeyama. CDX, Chinese Dai in Xishuangbanna; CHB, Han Chinese in Beijing; CHS, Southern Han Chinese; JPT, Japanese in Tokyo; KHV, Kinh in Ho Chi Minh City.

The principal component analysis (PCA) plot of 1,098 individuals obtained by OBi project using 491,109 SNPs that passed the quality control criteria formed several genetic clusters for individuals from the Miyako Islands, Okinawajima, and Japanese Archipelago (Hondo) (fig. 2). We plotted individuals having four grandparents born in the same place, 834, 32, 2, and 21 from the Miyako Islands, Okinawajima, Yaeyama Islands, and Hondo respectively, to infer the genetic components of each cluster, and we found that these clusters corresponded to people from the Japanese Archipelago (Hondo), people from the Okinawajima and people from the Miyako Islands (fig. 2). In this analysis, we found that the Miyako cluster was widely scattered along the PC1 axis compared with other clusters. Although the wide distribution of the Miyako cluster in the PC1 might be due to uneven sample size among populations, it is suggested that there is genetic heterogeneity within the Miyako populations. We also performed PCA using genotype data of 183,812 SNPs for 1,096 individuals living in the Ryukyu Archipelago along with the data for East Asians in the 1000 genomes database (*n* = 498), aboriginal Taiwanese (Ami and Atayal), and ancient Japanese (Jomon) (fig. 3). In this analysis, the Ryukyu cluster, including Okinawajima, Yaeyama, and Miyako, was located between Hondo (JPT and Hondo) and a Jomon individual, and aboriginal Taiwanese were plotted beyond the Hondo from the Ryukyu cluster, as reported previously (Sato et al. 2014). We observed that the Miyako cluster was widely scattered also in this analysis.

**Fig. 4.**
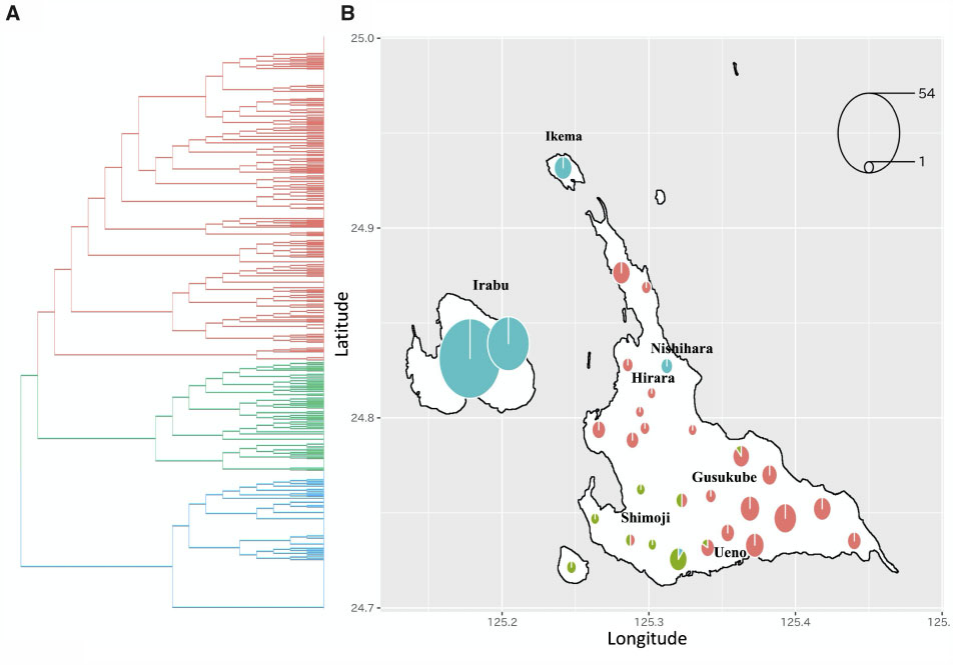
FineSTRUCTURE analysis with geographic information. (*A*) Dendrogram generated by the FineSTRUCTURE program. The Miyako population (834 individuals) was divided into three monophylotic subpopulations denoted by red, green, and blue composed of 437, 160, and 201 individuals, respectively. (*B*) Results of the FineSTRUCTURE analysis were mapped to the birthplaces of grandparents. Detailed locations of each settlement are listed in supplementary table 3, Supplementary Material online. The size of each pie chart is normalized from minimum (1) to maximum (54) (see pie chart legend at upper right) to represent the number of individuals derived from each focal location.

To elucidate the genetic population structure within the Miyako people in more detail, we conducted further analysis on 834 individuals having all four grandparents born in the Miyako Islands using PCA and Admixture analysis (supplementary figs. 1 and 2 and supplementary information A, Supplementary Material online). Considering haplotype information, we performed FineSTRUCTURE analysis, which more sensitively detects genetic differences. For example, the Tarama population is embedded in the Miyakojima cluster along with Gusukube, Hirara, Shimoji, and Ueno by PCA based on individual genome-wide SNPs (supplementary fig. 2, Supplementary Material online), but is located outside of the Miyakojima cluster by the haplotype-based PCA (supplementary fig. 3, Supplementary Material online). The dendrogram generated by the FineSTRUCTURE program shows that the Miyako population is divided into three subpopulations corresponding to geographic regions in the Miyako Islands (Miyakojima northeast, Miyakojima southwest, and Irabu/Ikema), although the signal continuity of haplotype structures exists (fig. 4*A* and supplementary figs. 4 and 5, Supplementary Material online). At first, the Miyako population is divided into two population groups, Miyakojima and Irabu/Ikema, which excludes one local population, Nishihara that belongs to the Hirara region in Miyakojima (fig. 4*B*). Further, individuals from Miyakojima are divided into two local subpopulations (fig. 4*B*): northeast (Hirara and Gusukube) and southwest (Shimoji and Ueno). The *F*_ST_ values between Miyakojima and Irabu/Ikema subpopulations were larger than those between two local subpopulations in Miyakojima (*F*_ST_ ± SE = 6.79 × 10^−3^ ± 0.017 × 10^−3^ and 8.74 × 10^−3^ ± 0.0225 × 10^−3^, Miyakojima northeast vs. Ikema/Irabu and Miyakojima southwest vs. Irabu/Ikema, respectively. *F*_ST_ ± SE = 1.64 × 10^−3^ ± 0.00729 × 10^−3^, Miyakojima northeast vs. Miyakojima southwest, table 1).

**Table 1.** Calculated *F*_ST_ among the Miyako Subpopulations.

Population 1	Population 2	*F*_ST_	SE
Miyakojima northeast	Irabu/Ikema	6.79 × 10^−3^	0.0170 × 10^−3^
Miyakojima southwest	Irabu/Ikema	8.74 × 10^−3^	0.0225 × 10^−3^
Miyakojima northeast	Miyakojima southwest	1.64 × 10^−3^	0.00729 × 10^−3^
Okinawajima	Irabu/Ikema	10.7 × 10^−3^	0.0365 × 10^−3^
Okinawajima	Miyakojima northeast	3.65 × 10^−3^	0.0216 × 10^−3^
Okinawajima	Miyakojima southwest	4.13 × 10^−3^	0.0244 × 10^−3^
Hondo	Irabu/Ikema	13.3 × 10^−3^	0.0487 × 10^−3^
Hondo	Miyakojima northeast	6.76 × 10^−3^	0.0348 × 10^−3^
Hondo	Miyakojima southwest	7.27 × 10^−3^	0.0379 × 10^−3^
Hondo	Okinawajima	3.57 × 10^−3^	0.0416 × 10^−3^

### Recent Demographic Histories for Each Subpopulation

**Fig. 5.**
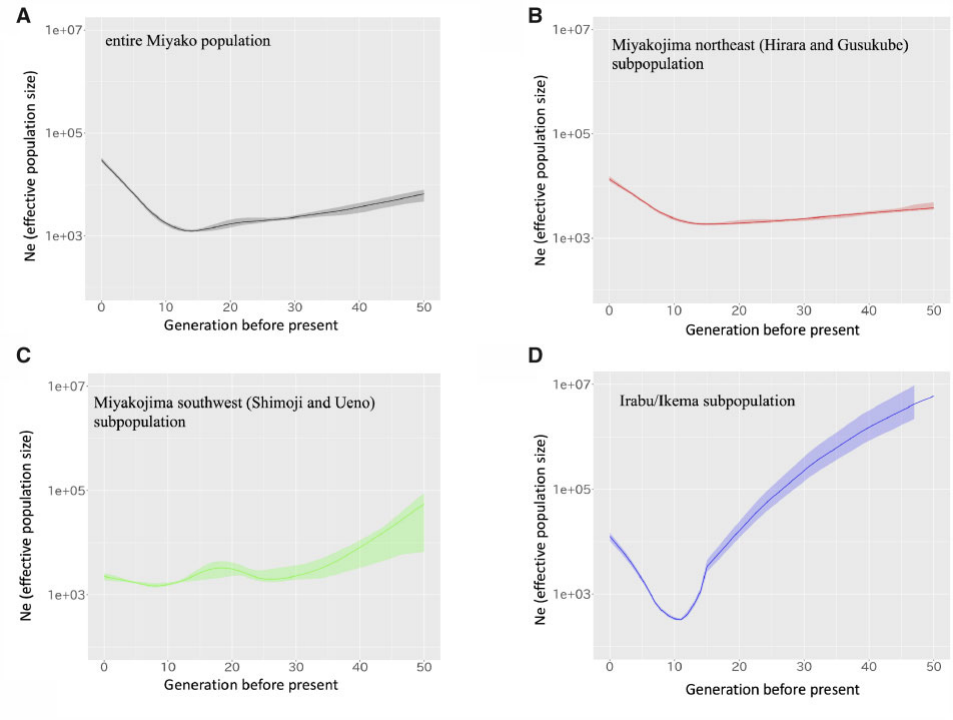
Demographic histories inferred by IBDseq and IBDNe. Changes in effective population sizes were estimated based on IBD segments. The vertical axis represents population size with a common logarithmic scale. The horizontal axis represents generation times. We estimated population sizes of (*A*) the entire Miyako population, (*B*) the Miyakojima northeast (Hirara and Gusukube) subpopulation, (*C*) the Miyakojima southwest (Shimoji and Ueno) subpopulation, and (*D*) the Irabu/Ikema subpopulation.

We inferred recent demographic histories of the Miyako population using the IBDNe program, which can estimate the change of a recent effective population size using information of shared identity by descent (IBD) segments (fig. 5 and supplementary figs. 6 and 7, Supplementary Material online). To eliminate the possibility for program-specific artificial error, such as breaks and short gaps in IBD segments, we used three different methods for IBD detection (IBD-seq, refined IBD, and GERMLINE) and obtained consistent estimation results from the three methods. First, we applied this analysis to all 834 individuals derived from the Miyako Islands, and we observed that the effective population size had gradually increased, except for around 10–15 generations ago (fig. 5*A*). Then, we also performed this analysis using the three subpopulations defined by FineSTRUCTURE analysis. One subpopulation derived from Miyakojima northeast (Hirara and Gusukube) showed similar demographic history with all Miyako populations (fig. 5*B*). However, the other two subpopulations showed a bottleneck of the effective population size around ten generations ago (fig. 5*C* and *D*). In particular, the Irabu/Ikema subpopulation seemed to have experienced a drastic decrease in the effective population size during this period.

### Admixture from Other Populations to Miyako Islands

We examined the haplotype sharing profiles between each Miyako subpopulation and populations from the 1000 genomes project (supplementary fig. 8, Supplementary Material online). Among 26 populations from the 1000 genomes project, JPT had the largest number of haplotypes shared with the Miyako subpopulations. Other East Asian populations also shared a large portion of haplotypes with the Miyako populations. When we compared the Miyako subpopulation with the 1000 genomes data, Miyakojima northeast always shared the largest number of haplotypes with other populations, whereas shared haplotypes between Irabu/Ikema and other populations were always smallest among the Miyako subpopulations.

**Fig. 6.**
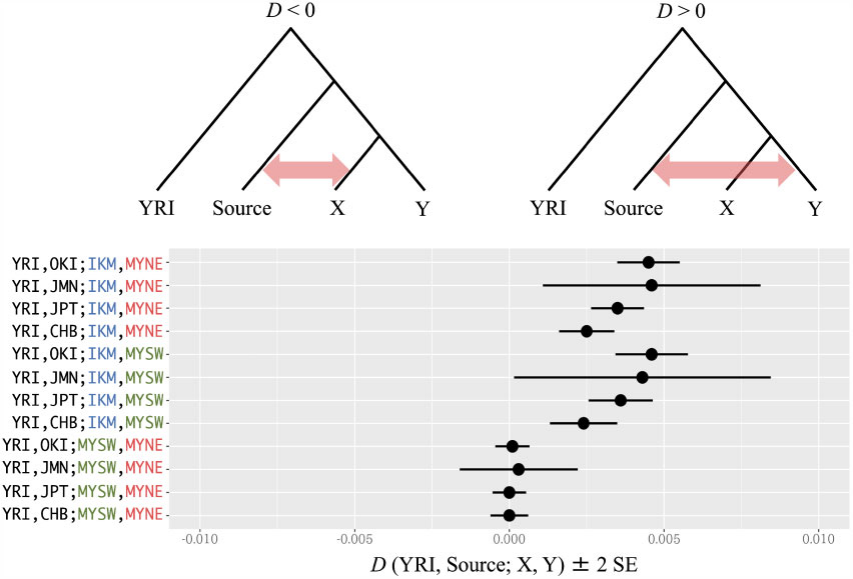
The genetic affinity between Miyako and other populations. Patterson’s *D* statics of each topology was shown with ±2 SE. We used JPT, JMN, OKI, and CHB as source population, and YRI as outgroup. IKM, Irabu/Ikema; MYNE, Miyakojima northeast; MYSW, Miyakojima southwest; JMN, Jomon; JPT, Japanese in Tokyo; OKI, Okinawajima; YRI, Yoruba in Ibadan, Nigeria; CHB, Han Chinese in Beijing.

Patterson’s *D* statistics provide information about gene flow among the focal populations. We tested *D* (YRI, source; X, Y) for JPT, CHB, Okinawajima, or Jomon as the source population (fig. 6). When we set X = Irabu/Ikema and Y = Miyakojima northeast or Miyakojima southwest, the *Z* scores were always significantly positive (*Z* score > 2). Among them, the Z score of *D* (YRI, Okinawajima; Irabu/Ikema, Miyakojima southwest) was the highest (supplementary table 2, Supplementary Material online). *D* statistics using two Miyakojima subpopulations were always nearly zero, regardless of the source population. Thus, it is suggested that the Miyakojima subpopulations experienced different gene flows against Irabu/Ikema subpopulation.

### Migration among Local Subpopulations in Miyako Islands

**Fig. 7.**
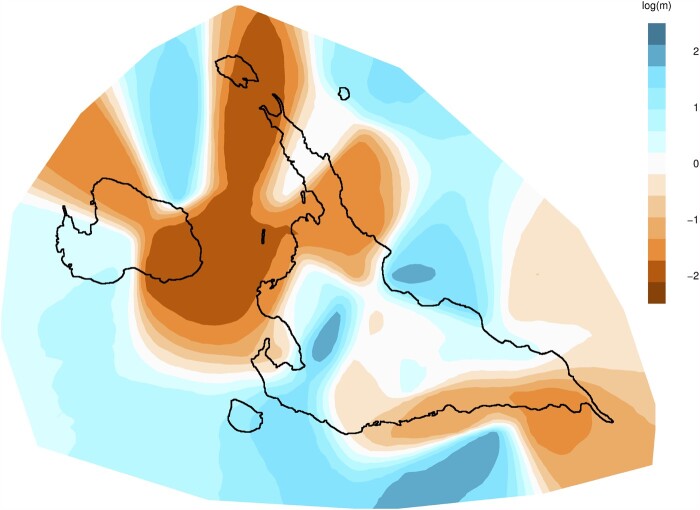
Distribution of effective migration surface. We estimated the EEMS rates within the Miyako Islands using 240 individuals. Blue regions indicate higher effective migration, whereas orange indicates lower effective migration.

Migration among the Miyako Islands was estimated using both genotyping and geographic data. The Estimating Effective Migration Surfaces (EEMS) program can estimate effective migrations between neighboring demes based on the stepping-stone model (fig. 7). Since Markov Chain Monte Carlo (MCMC) runs of the EEMS program had converged (supplementary fig. 9, Supplementary Material online), this analysis is considered to be reliable. We found that the populations of Ikema, Irabu, and northern Miyakojima showed low effective migration, suggesting that genetic similarities tended to decay faster in these regions. Similarly, southern Miyakojima also showed low effective migration. In contrast, the central region of Miyakojima showed relatively high effective migration, suggesting that genetic similarities tended to decay slowly. The result of isolation by distance analysis suggests that the contribution of geographic distance to genetic differentiation in the Miyako populations is very weak (supplementary fig. 10, Supplementary Material online, see supplementary information B, Supplementary Material online).

### Demographic Inferences

**Fig. 8.**
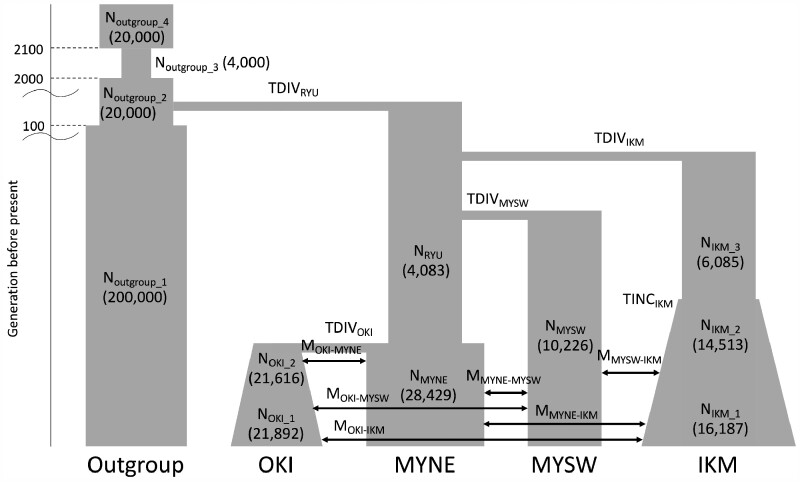
A proposed demographic model for fastsimcoal2 analysis. We proposed a demographic model considering the population size changes inferred from our haplotype-based analysis to estimate demographic parameters by fastsimcoal2. The fixed parameters related the outgroup population (CHB) and point estimations of population sizes for individual populations are shown in parentheses. CHB, Han Chinese in Beijing; IKM, Irabu/Ikema; MYNE, Miyakojima northeast; MYSW, Miyakojima southwest; OKI, Okinawajima.

Assuming a demographic model, we estimated the parameter values and their 95% confidence intervals (CI) by a coalescent simulation using fastsimcoal2 (fig. 8 and table 2). In the proposed model, the Irabu/Ikema population firstly split away from the ancestral Ryukyu population about 41 generations ago (TDIV_IKM_, 95% CI: 38–57), followed by a split of the Miyakojima southwest population from the ancestral Ryukyu population about 16 generations ago (TDIV_MYSW_, 95% CI: 10–27), and the differentiation of the ancestral Ryukyu population into two populations (Miyakojima northeast and Okinawajima populations) about seven generations ago (TDIV_OKI_, 95% CI: 2–18). Since, the 95% CI for the point estimation of TDIV_IKM_ did not overlap with that of any other point estimation, namely TDIV_OKI_, TDIV_MYSW_, we thought the order of the split event for the Irabu/Ikema population from the ancestral Ryukyu population was confirmed to be the most ancient. In contrast, the 95% CIs of the latter two split times, TDIV_OKI_ and TDIV_MYSW_, were found to overlap, and we were unable to confirm the order of these two events exactly. Although we induced epochs for the population size change in the Irabu/Ikema population in this simulation, we did not observe the population bottleneck identified in the IBDNe analyses. Instead, an increase in the population size was observed about ten generations ago (TINC_IKM_, 95% CI: 1–19). Furthermore, we assumed recent migrations among the Ryukyu populations, and among them, a parameter for the migration between Okinawajima and Miyako northeast showed the highest in point estimation (M_OKI-MYNE_ = 3.44 × 10^−4^; 95% CI: 6.64 × 10^−9^–9.44 × 10^−3^).

**Table 2.** Estimated Demographic Parameters with 95% CIs by fastsimcoal2.

Parameter	Point Estimation	95% CI
TDIV_OKI_	7	2–18
TDIV_MYSW_	16	10–27
TDIV_IKM_	41	38–57
TDIV_RYU_	109	104–125
TINC_IKM_	10	1–19
N_OKI_1_	21,892	20,271–27,770
N_OKI_2_	21,616	15,435–27,415
N_MYNE_	28,429	20,840–37,602
N_MYSW_	10,226	3,156–15,804
N_IKM_1_	16,187	5,168–16,750
N_IKM_2_	14,513	3,775–17,496
N_IKM_3_	6,085	4,277–15,420
N_RYU_	4,083	3,708–4,939
M_OKI-MYNE_	3.44 × 10^−4^	6.64 × 10^−9^–9.44 × 10^−3^
M_OKI-MYSW_	8.95 × 10^−5^	5.22 × 10^−9^–3.63 × 10^−2^
M_OKI-IKM_	7.09 × 10^−8^	2.58 × 10^−10^–1.05 × 10^−2^
M_MYNE-MYSW_	4.22 × 10^−6^	2.96 × 10^−9^–5.78 × 10^−2^
M_MYNE-IKM_	1.47 × 10^−6^	2.06 × 10^−9^–3.47 × 10^−3^
M_MYSW-IKM_	1.64 × 10^−9^	5.55 × 10^−10^–1.05 × 10^−3^

## Discussion

Based on haplotype information collected through the OBi Project, we have demonstrated the fine-scale genetic structure of the Miyako Islands for the first time. Although previous studies clarified the genetic differences between the Hondo and Ryukyu people (Yamaguchi-Kabata et al. 2008; HUGO Pan-Asian SNP Consortium 2009; Okada et al. 2018) and differences within the Ryukyu Archipelago, such as the Okinawajima, Miyako, and Yaeyama Islands (Matsukusa et al. 2010; Sato et al. 2014), the detailed population structures within the Miyako Islands have not yet been elucidated. We collected genome-wide genotyping data for over 1,000 individuals which covered most regions of the Miyako Islands. These data make it possible to infer the detailed genetic structure and the recent demographic history of the Miyako Islands.

Our results from FineSTRUCTURE analysis and PCA suggest that a significant genetic heterogeneity exists among people living in the Miyako Islands (fig. 4 and supplementary figs. 3–5, Supplementary Material online). To the best of our knowledge, our report is the first to show the presence of subpopulations within such a small island group (about 204 km^2^), although the signal continuity of haplotype structures exists and absolute genetic differentiation among these subpopulations, especially two Miyakojima subpopulations is very low (table 1). This genetic diversity may reflect the population history of the Miyako people. Until 1902, free migration within the Miyako Islands was restricted by the implementation of the capitation tax system (Miyakojima City Board of Education 2012), which may have enhanced genetic diversity within the Miyako Islands.

Our analyses showed that the Miyako people are genetically divided into three subpopulations. Until 2005, Miyakojima had four local districts (Gusukube, Hirara, Shimoji, and Ueno), which were merged to the Miyakojima government. Because the genetic distances among the different subpopulations were nearly correlated with geographic distances, we defined the three subpopulations as Miyakojima northeast (Hirara and Gusukube), Miyakojima southwest (Shimoji and Ueno), and Irabu/Ikema subpopulations. The exception is the Nishihara settlement in the Hirara locality which does not belong to the Miyakojima northeast subpopulation but rather to the Irabu/Ikema subpopulation.

Based on information in the literature, the Nishihara settlement was established by immigrants from Ikema Island in 1873 (Miyakojima City Board of Education 2012). Thus, our genetic analysis results were consistent with historical documentation, indicating the high reliability of our analyses. Interestingly, the results of our genetic analysis are consistent with the current distribution of the Miyako language. The traditional Miyako language is one of the dialects of Ryukyu languages and has great diversity depending on the regional populations within the Miyako Islands (Shimoji and Pellard 2010). Although most of localities in Miyako Islands have specific dialects, it has been shown that a dialect for the Nishihara settlement is almost the same with that in the Ikema island (Takubo 2017).

By IBDNe program, we estimated the current effective population sizes for all the Miyako Islands, Irabu/Ikema, Miyakojima northeast, and Miyakojima southwest as 29,800, 12,600, 13,600, and 2,260, respectively. Since recent census population size of the Miyako Islands is about 55,000 and estimated effective population size of all Miyako population is broadly agree to the recent census population size. However, it has been reported that a strong population bottleneck event might influence the accuracy of the estimated population size at older generation periods in IBDNe program: The information for the remaining few haplotypes after the bottleneck event is insufficient for accurate estimation for population sizes before the bottleneck event (Browning et al. 2018). We further calculated the total length of runs of homozygosity (ROH) for each subpopulation to infer recent population histories (supplementary fig. 11, Supplementary Material online). The results indicate that the ROH of the Irabu/Ikema subpopulation was significantly longer than those of the other two subpopulations (Tukey’s test, *P *<* *0.001), providing further evidence for a specific population bottleneck in the Irabu/Ikema subpopulation.

The Miyakojima subpopulations experienced a different history against Irabu/Ikema subpopulation. We showed that the Miyakojima subpopulations did not experienced any drastic population bottlenecks (supplementary fig. 7, Supplementary Material online). The calculated *D* statistics indicate that there is little evidence for the gene flow from other populations to the Irabu/Ikema subpopulation, whereas the Miyakojima subpopulations showed signatures of migration from other populations (fig. 6), suggesting that the Irabu/Ikema subpopulation received gene flows that were different from those of the Miyakojima subpopulations. Although *D* (YRI, Okinawajima; Irabu/Ikema, Miyakojima southwest) showed the highest Z score, we could not clarify the source population of this gene flow. However, considering the results of demographic inferences, immigration from the Northern islands, such as Okinawajima, to Miyakojima islands might be related, at least in part, to the historically attested immigration during the Gusuku period (900 − 500 BP).

Although there are several possible explanations for the bottleneck in the Irabu/Ikema subpopulation, one remarkable disaster during this period is a strong candidate. A large earthquake and a subsequent tsunami hit the Yaeyama and Miyako Islands in 1771 (the Great Tsunami of Meiwa) (Makino 1968). More than 2,000 people were killed by this disaster in the Miyako Islands. In addition, there were large migrations from Ikema to Miyakojima after this disaster. Since many people on Miyakojima also died in the tsunami, it was difficult for several settlements to keep city functions, so the government forced people to move from Ikema to Miyakojima. It is possible that these events were involved in the genetic bottleneck we found in our data. Although the documentation described that both southwest Miyakojima and Ikema had been critically damaged (Miyakojima City Board of Education 2012), our analysis showed a large genetic bottleneck only in the Irabu/Ikema subpopulation; therefore, we need to obtain more historical and genetic information to clarify this inconsistency.

Our coalescent-based demographic inference suggested three waves of migrations into the Miyako Islands. The oldest migration wave may have generated the ancestral Irabu/Ikema population (41 generations ago), followed by two more recent migrations, which occurred at closer intervals (16 and 7 generations ago). Considering the archaeological evidence and the results of haplotype sharing and *D* statistics analyses, the time of migration into Irabu/Ikema may date back to the Gusuku period (900 − 500 BP) as described in the introduction (supplementary table 1, Supplementary Material online), whereas the two more recent migrations into Miyakojima may have occurred during the Ryukyu Kingdom period. These migrations influence the current population genetic structure of the Miyako Islands.

The fine-scale genetic structure information that we identified is important for performing genetic association studies for the Ryukyu people. Genome-wide polygenic risk scores (PRS) constructed from large-scale GWAS data (sample size of hundreds of thousands to a million) could precisely predict the onset of several common diseases in people of European or East Asian descent (Torkamani et al. 2018; Sakaue et al. 2020). Aside from having highly qualified PRS in European populations, accurate PRS have not been available for other ethnic groups (Kerminen et al. 2019; Martin et al. 2019), including the Ryukyu people, because sufficiently powered GWAS has not been performed and there is regional genetic heterogeneity among local regional populations in the Ryukyu people. In addition, some diseases, that is, human T-cell lymphotropic virus type 1 and nonacquired immune deficiency syndrome-associated Kaposi’s sarcoma, are more frequently observed in the Ryukyu Archipelago than on other islands in Japan (Ishida et al. 1985; Awazawa et al. 2017; Koganebuchi and Kimura 2019), likely because of their unique genetic backgrounds. However, the relationship between disease risk and regional differences in genetic background remains unknown. Therefore, further investigation is required to understand the genetic architecture of the Ryukyu people and to obtain the useful genetic information for improving the medical healthcare of the Ryukyu populations.

In this study, we produced genetic evidence that reveals the previous population isolation history and gene flow among people living in the Miyako Islands. The genetic structure we observed likely reflects past events experienced in the Miyako Islands, such as migrations and disasters. We aim to accumulate more genetic information derived from the Ryukyu Archipelago with clinical information through the OBi Project, and such data will enable us to further understand the population history of the Ryukyu Archipelago and to identify novel genetic factors related to susceptibility to several common and/or rare diseases.

## Materials and Methods

### Participants, DNA Extractions, and Genotyping

Saliva or blood samples were collected from 1,240 individuals living in the Miyako Islands between 2016 and 2017 as part of the OBi Project. All participants gave written informed consent before their enrollment in the study. For the survey of the origin of individual participants, we obtained information of the birthplace (islands) for their four grandparents by a questionnaire. If their birthplaces were Miyakojima or Irabu, we asked for more detailed information regarding birth areas on each island dating as far back as possible. Genomic DNA was extracted from saliva or blood samples, and we genotyped 665,326 SNPs using the Asian Screening Array (Illumina, San Diego, CA). The protocol of this study was approved by the Ethics Committees at University of the Ryukyus (approval number 241).

### Quality Control

The criteria for including SNPs in our experiments were as follows: 1) SNP calling rates were 98% or higher, 2) individual calling rates were 98% or higher, 3) genotype distributions were in accordance with Hardy–Weinberg equilibrium (*P *>* *10^−6^), and 4) SNPs were polymorphic in the focal population (minor-allele frequency > 0). In addition, individuals having a shared identity-by-descent ($\hat{\pi }$) higher than 0.25 were excluded. As a result, 1,098 individuals with 491,109 SNPs passed these quality controls and were used in subsequent analyses. We carried out PCA to evaluate the population structure of these samples. These analyses were performed using PLINK 1.9 (Chang et al. 2015).

### Inferring the Population Genetic Structure between the Miyako and Other Populations

We compared our genotyping data with those of the other available populations. We obtained the following genotyping data for comparison: East Asian populations of the 1000 Genomes Project Phase 3 (1000 Genomes Project Consortium 2012) (ftp://ftp.1000genomes.ebi.ac.uk/vol1/ftp/), aboriginal Taiwanese (Ami and Atayal) from the Simon Genome diversity Project (Mallick et al. 2016) (http://sharehost.hms.harvard.edu/genetics/reich_lab/dgdp), and Jomon F23 individual (Kanzawa-Kiriyama et al. 2019). We merged these genotyping data with our OBi data and filtered them using the same quality control criteria (see previous section in detail). After filtering, 1,597 individuals with 183,812 SNPs were remained. These data were utilized for PCA analysis using PLINK 1.9.

### Inferring the Population Genetic Structure within the Miyako Islands

To infer a more detailed population structure in the Miyako Islands, 834 individuals having all four grandparents born in the Miyako Islands were included for further analyses. We carried out PCA using these 834 individuals and confirmed that there are no population outliers among the 834 individuals (PC1 and PC2 values of all samples were within mean ± 6 SD). To infer the population structure in greater detail, we used the haplotype-based clustering method in the FineSTRUCTURE/ChromoPainter program (v4.0.1) in which individuals were assigned into genetically homogenous groups using a nonparametric Bayesian mixture model implemented through an MCMC algorithm (Lawson et al. 2012). Detailed methods are described in supplementary information C, Supplementary Material online.

### Estimating Demographic History Based on IBD

The recent demographic histories of the local populations were inferred based on the IBD segments. We reconstructed the IBD segments using IBD-seq ver: r1206 (Browning BL and Browning SR 2013a). This method detects shared IBD segments among individuals from unphased sequence data. Additionally, GERMLINE (Gusev et al. 2008) and refined IBD (Browning BL and Browning SR 2013b) were used for IBD detection. Since these programs used phased genotyping data as input, we prepared them by EAGLE v2.4.1 (Loh et al. 2016). After IBD detection by refined IBD, we utilized the Java script “merge-ibd-segments.17Jan20.102.jar” to remove any breaks and short gaps in the IBD segments. Based on these IBD segments, we inferred recent demographic history using IBDNe ver.19Sep19 (Browning BL and Browning SR 2015), which estimated the change of effective population size from around four generations to around 50 generations ago for SNP array data. Because the recommended IBD-length threshold was more than 4 cM for the SNP array data in the IBDNe analysis, we set 6 cM as the threshold of our analysis. These analyses were applied to all filtered genotype data (834 individuals) and three subpopulations (437, 160, and 201 individuals for Miyakojima northeast, Miyakojima southwest, and Irabu/Ikema, respectively). We additionally carried out IBDNe analysis using a 2-subpopulation model.

### Haplotype Sharing

We estimated shared IBD segments between each subpopulation in the Miyako and other populations from the 1000 genomes project using the IBD-seq program. The physical lengths of each IBD segment (in base pairs) were converted into genetic distances (in centimorgans, cM) by extrapolating the genetic map provided by HapMap (International HapMap Consortium 2005).

To compare haplotype sharing between the two populations, we calculated the haplotype sharing index of each population pair using the following equation (Atzmon et al. 2010):

$${\mathrm{IBD}}^{IJ}=\frac{\sum_{i=1}^{n}\sum_{j=1}^{m}{\mathrm{IBD}}^{ij}}{nm}\;\times \;100$$

where IBD^*ij*^ is the total sharing haplotype between individuals *i* and *j* from population *I* and *J*, respectively. We summarized the total IBD lengths of possible all pairs from two populations and computed the arithmetic mean of the shared segments for each pair of populations.

### Patterson’s *D* Statistics to Detect Gene Flow

We calculated the *D* statistics to infer whether the genetic differences among the Miyako subpopulations were derived from genetic drift or ancestry differences. We chose YRI as the outgroup and JPT, CHB, Okinawajima, and Jomon as the source populations of admixture. Each test was carried out using AdmixTools (Patterson et al. 2012). We compared the source populations with two Miyako subpopulations to know recent and ancient admixture from the source populations to each Miyako subpopulation.

### Estimating Effective Migration Surfaces

The migration and relative diversity among the populations in the Miyako Islands were estimated using EEMS ver.0.0.0.9000 (Petkova et al. 2016). This program was used to calculate the average pairwise distances between populations from genotyping data and geographic information using the bed2diffs function. We selected 240 individuals having all four grandparents with the same birthplace at detailed address level 2 (supplementary table 3, Supplementary Material online). We excluded individuals whose ancestors were born on Tarama or Minna, because these islands are located far from the other islands of the Miyako Islands. The 36 represented locations for each local address were used in the analysis. Detailed input data for EEMS analysis are listed in supplementary table 3, Supplementary Material online. We ran the EEMS MCMC model for a burn-in of 10,000,000 iterations and conducted sampling on 20,000,000 iterations. The number of demes was set to 200. The results were visualized using the rEEMSplot package provided by the EEMS program (R version 3.6.2).

### Demographic Inferences by fastsimcoal2

We chose four populations (Okinawajima, Miyakojima northeast, Miyakojima southwest, and Irabu/Ikema) along with CHB from the 1000 genomes project as an outgroup for the coalescent simulation to infer the demographic population history, including the effective population sizes, migration rates, and divergence times under the assumption of recent migrations among the Ryukyu populations. We proposed a demographic model considering the population size changes inferred from our haplotype-based analysis (fig. 8). Detailed methods are described in supplementary information D, Supplementary Material online.

## Supplementary Material

Supplementary data are available at *Molecular Biology and Evolution* online.

## Supplementary Material

msab005_Supplementary_Data
