# Supplementary material for: Fine-Scale Genetic Structure and Demographic History in the Miyako Islands of the Ryukyu Archipelago
Source: Mol Biol Evol. 2021 Jan 12;38(5):2045–56. doi: 10.1093/molbev/msab005 (PMC8097307; doi:10.1093/molbev/msab005)
Supplement: msab005_Supplementary_Data [file msab005_supplementary_data.zip › Miyako_SFigs_highResolution.pdf]

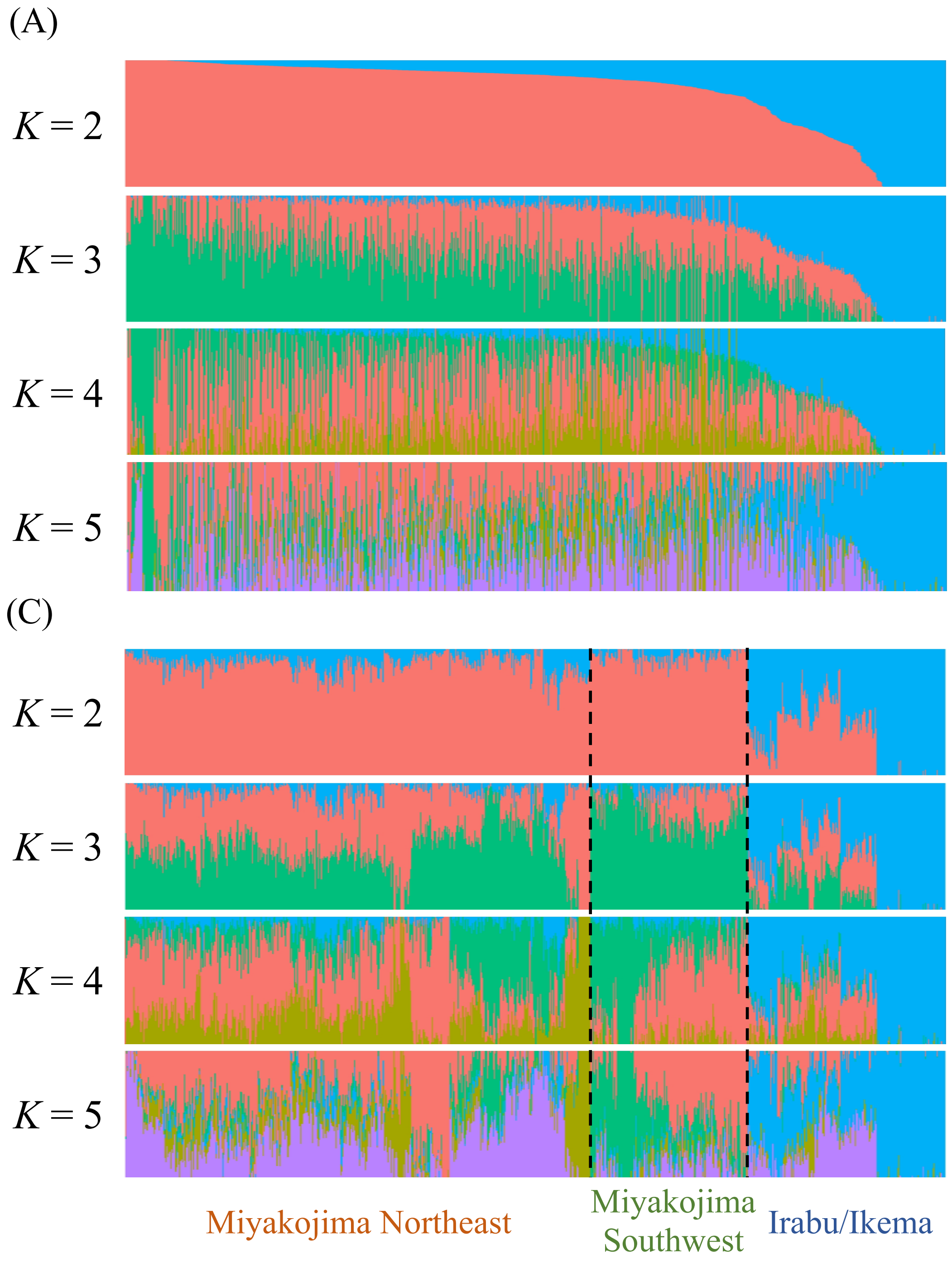

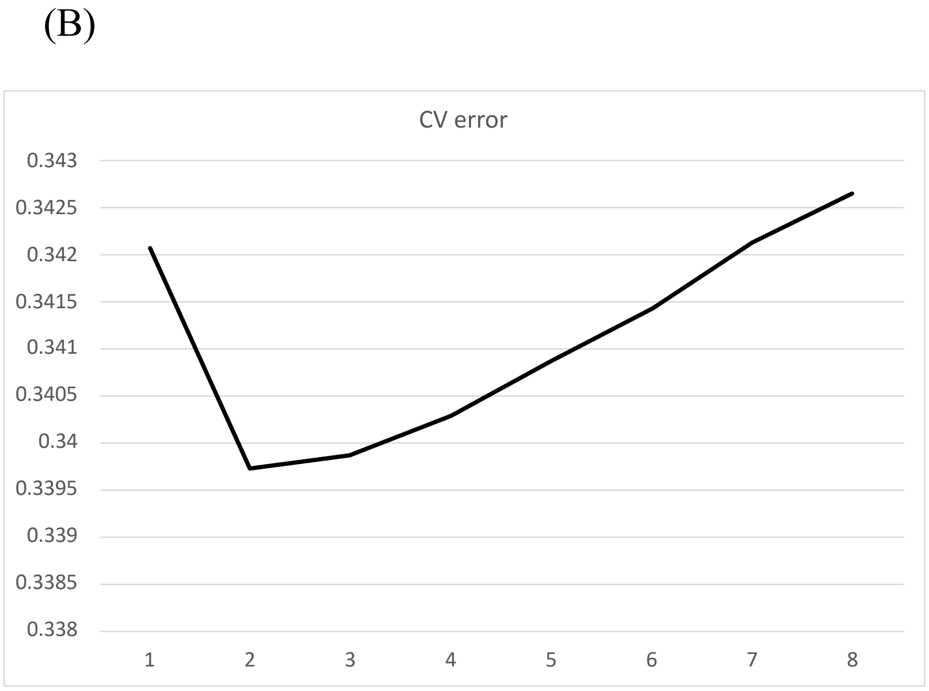

Supplementary Figure 1. Results of Admixture analysis
(A) Admixture plots from K = 2 to 5 are aligned along the result of K = 2. (B) The cross-validation error for each K value was calculated. (C) Individuals are aligned along the FineSTRUCTURE dendrogram.

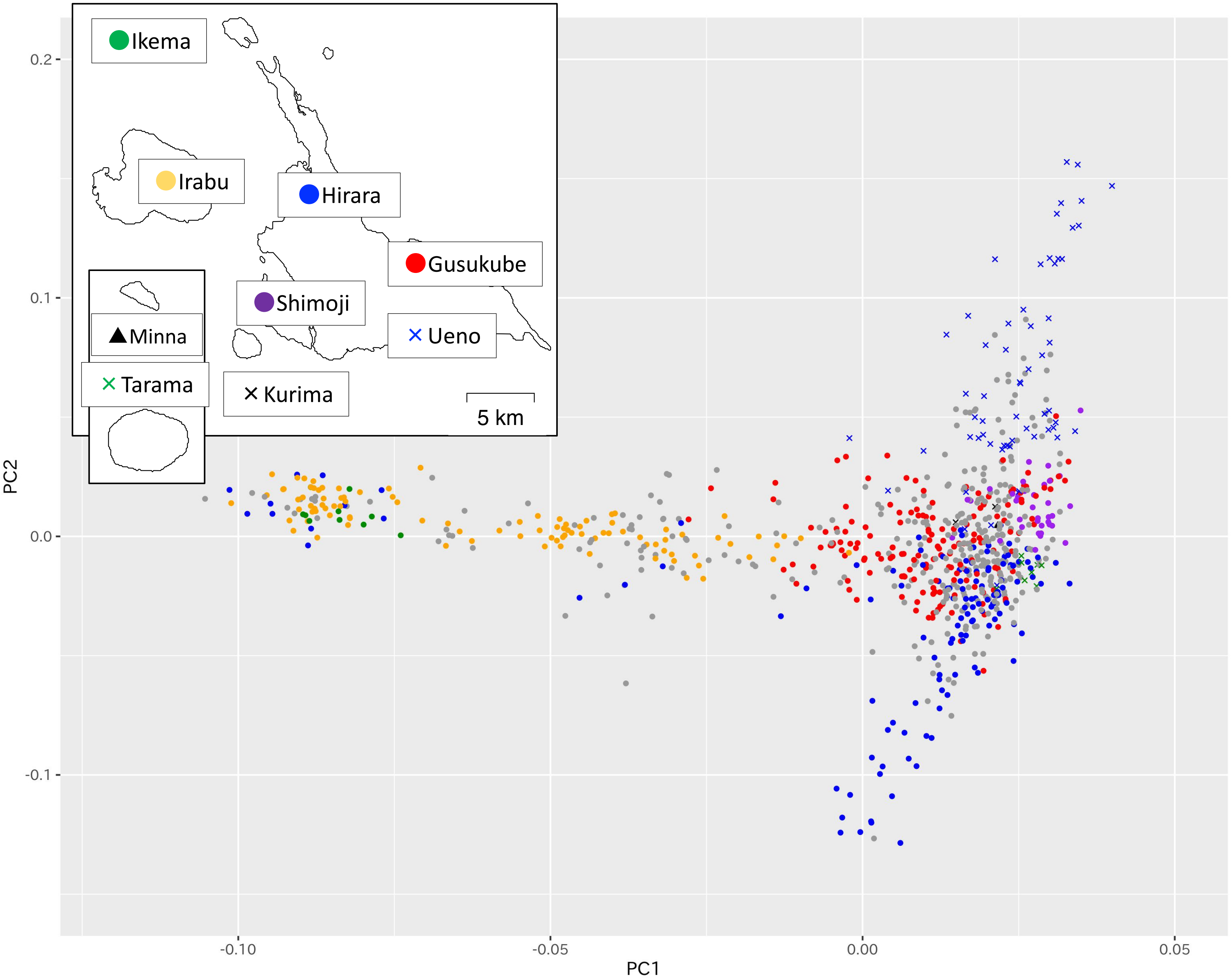

Supplementary Figure 2. PCA plot for populations from the Miyako Islands

PC values were calculated using 834 individuals having four grandparents derived from the Miyako Islands. Each symbol denotes each locality for the birthplace of the grandparents at detailed address level 1 (Supplementary Table 3) as indicated in the inset.

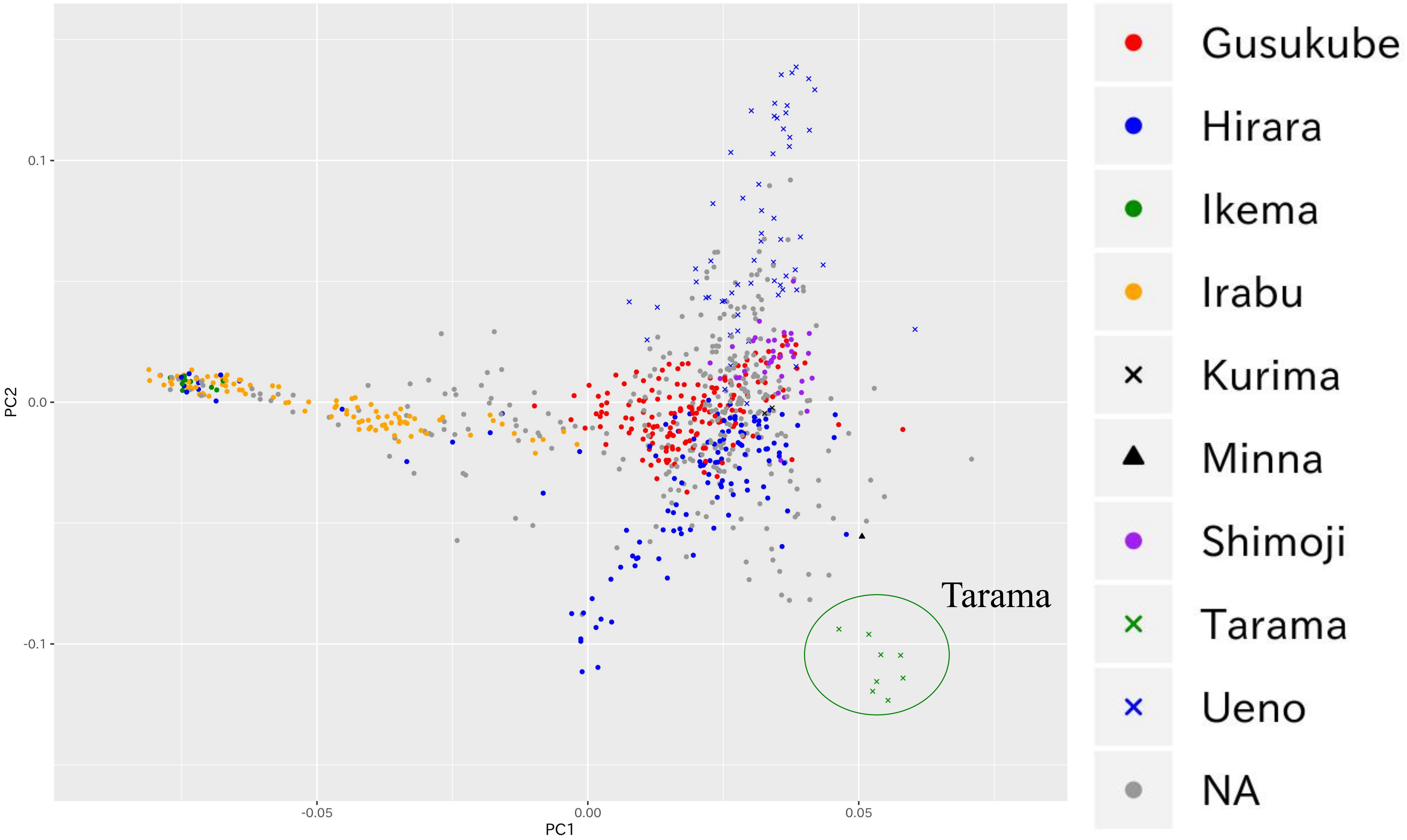

Supplementary Figure 3. Haplotype-based PCA plot for populations from the Miyako Islands divided by birthplace of grandparents
Haplotype-based PC values were calculated by the FineSTRUCTURE program using 834 individuals having four grandparents derived from the Miyako Islands. Each symbol denotes birthplace of the grandparents.

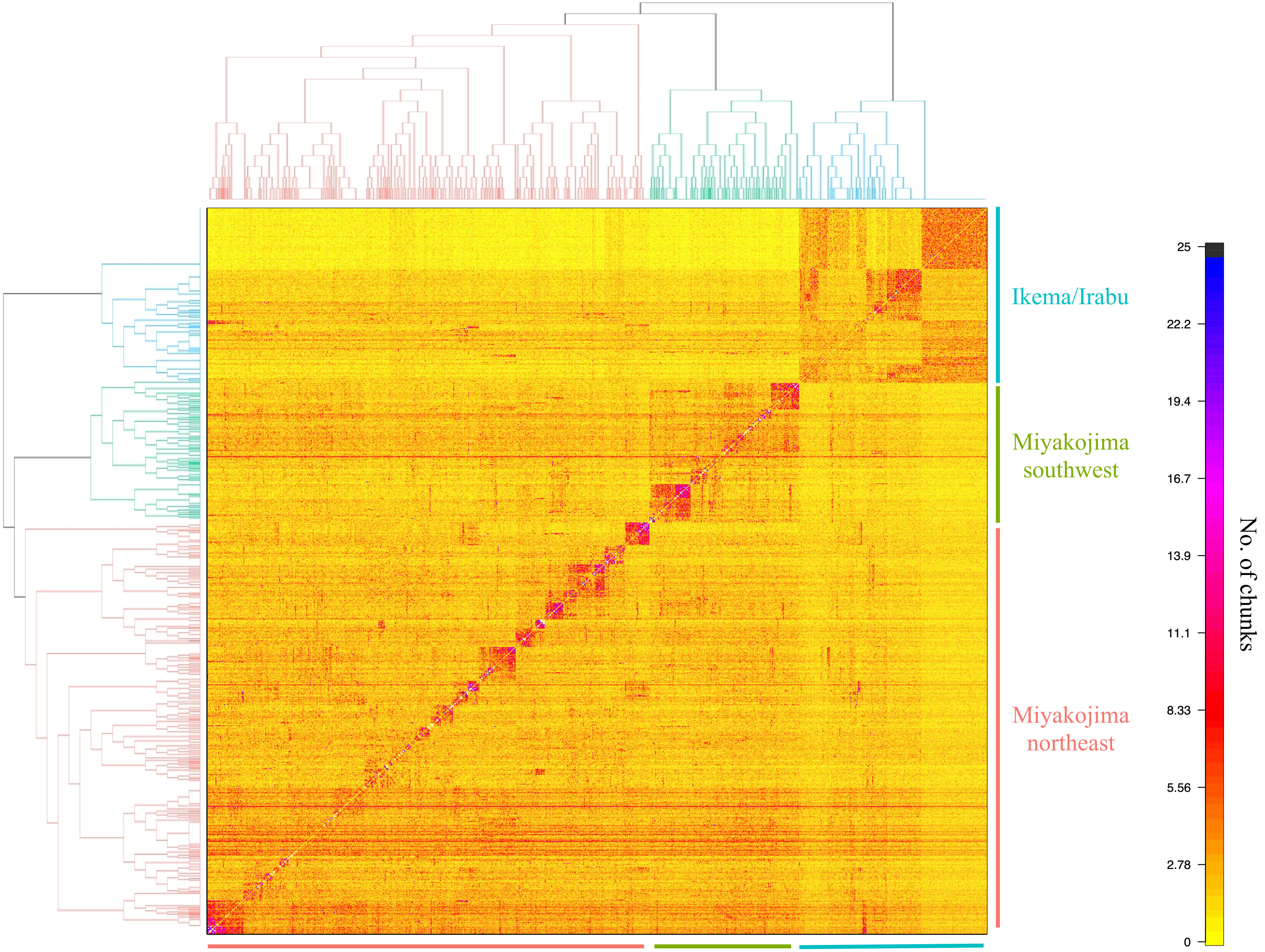

Supplementary Figure 4. Coancestry matrix of fineSTRUCTURE analysis

Each row of the heatmap represents the number of chunks shared between the recipient and every individual as a donor (columns). Intensity on the heatmap indicates the abundance of shared haplotypes. Each subpopulation is clustered and observed block-like patterns.

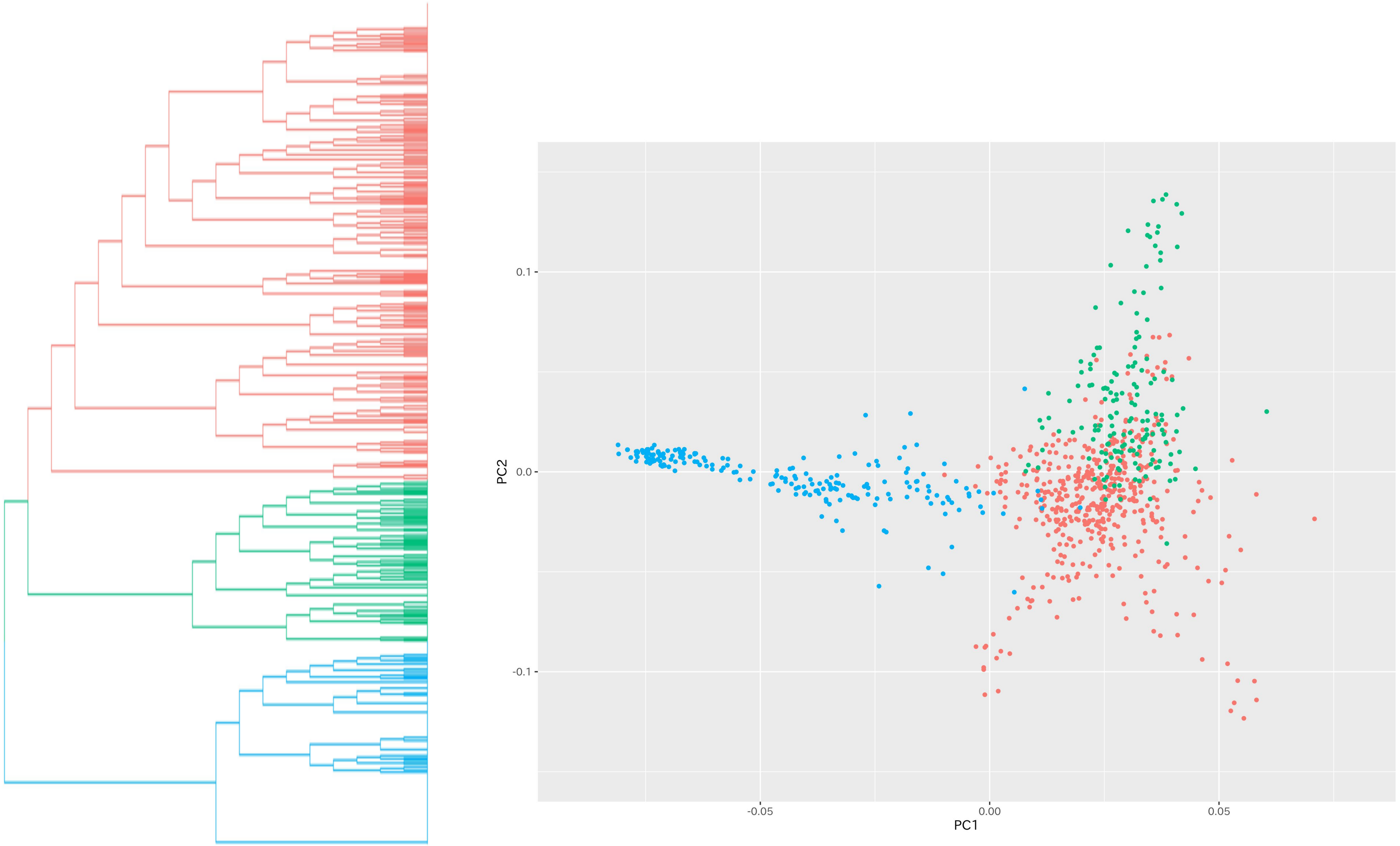

Supplementary Figure 5. Haplotype-based PCA plot for populations from the Miyako Islands divided by subpopulations
Haplotype-based PC values were calculated by the FineSTRUCTURE program using 834 individuals having four grandparents derived from the Miyako Islands. Each subpopulation defined by the FineSTRUCTURE program is denoted by a color as shown in the left panel.

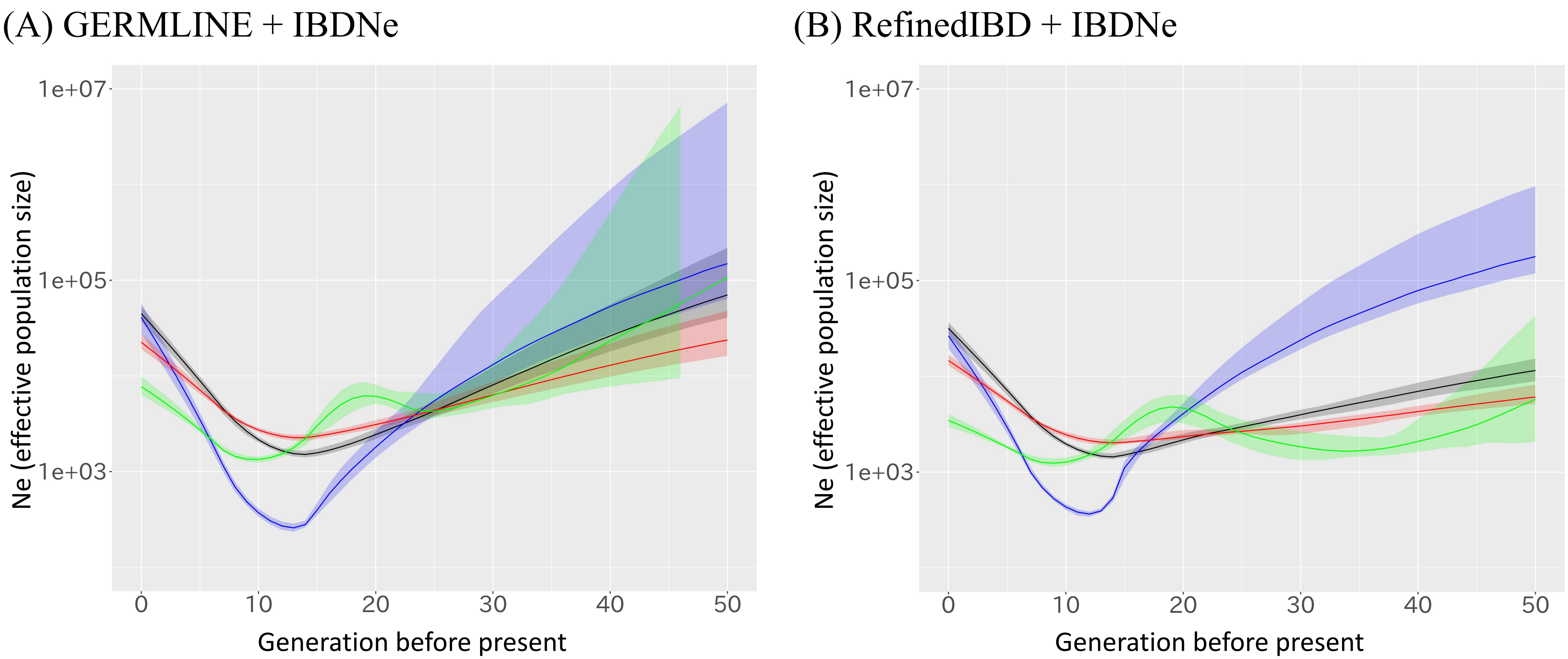

Supplementary Figure 6. Demographic histories inferred by refined IBD/ GERMLINE and IBDNe
Changes in effective population sizes were estimated based on IBD segments. The vertical axis represents population size with a common logarithmic scale. The horizontal axis represents generation times. We estimated population sizes of the entire Miyako population (black line), the Miyakojima northeast (Hirara and Gusukube) subpopulation (red line), the Miyakojima southwest (Shimoji and Ueno) subpopulation (green line), and the Irabu/Ikema subpopulation (blue line).

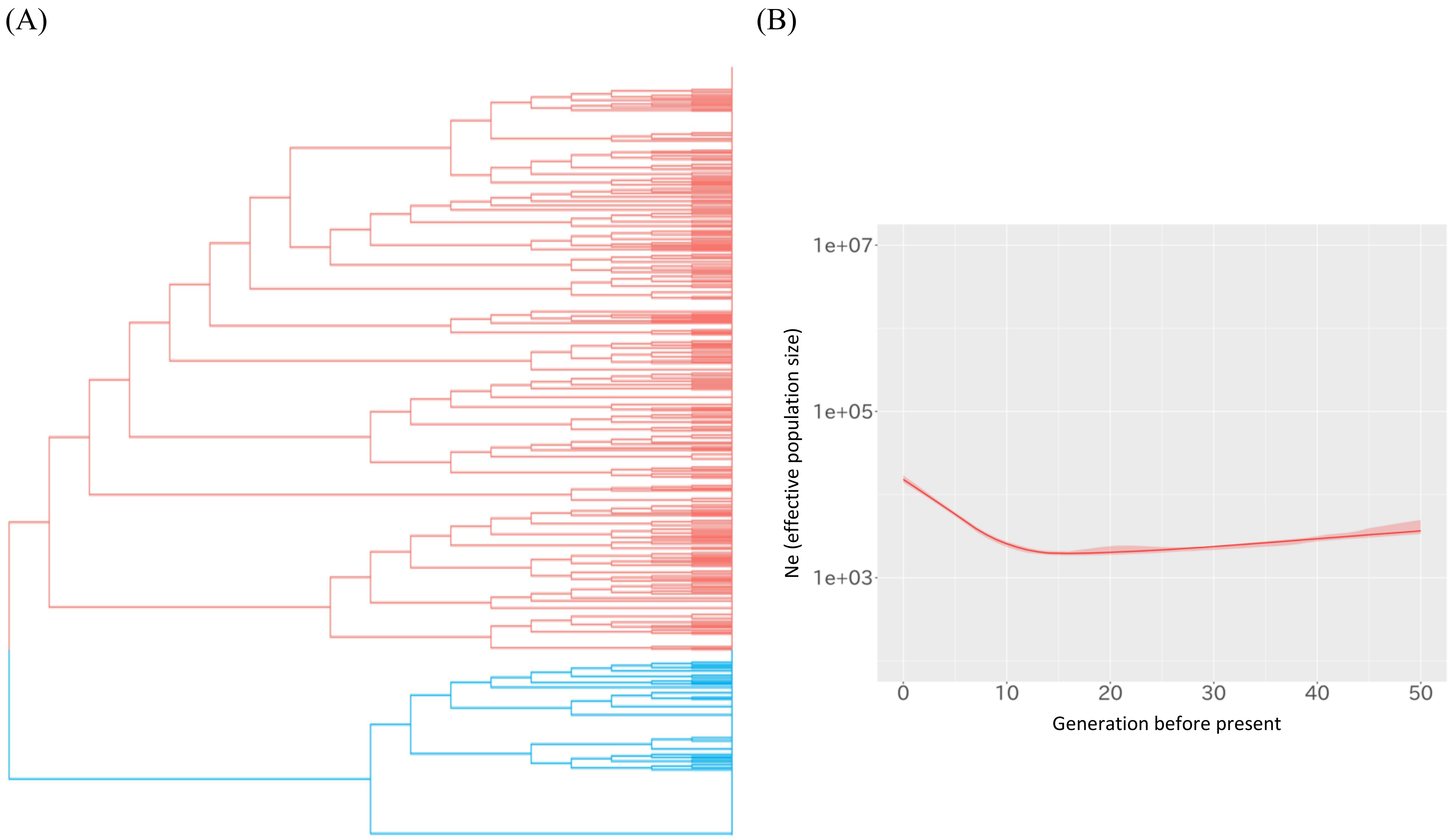

Supplementary Figure 7. Estimated demographic histories using two subpopulations

(A) FineSTRUCTURE dendrogram are divided into two subpopulations, Miyakojima (red) and Ikema/Irabu (blue). (B) demographic histories of Miyakojima subpopulation was estimated by IBDNe program.

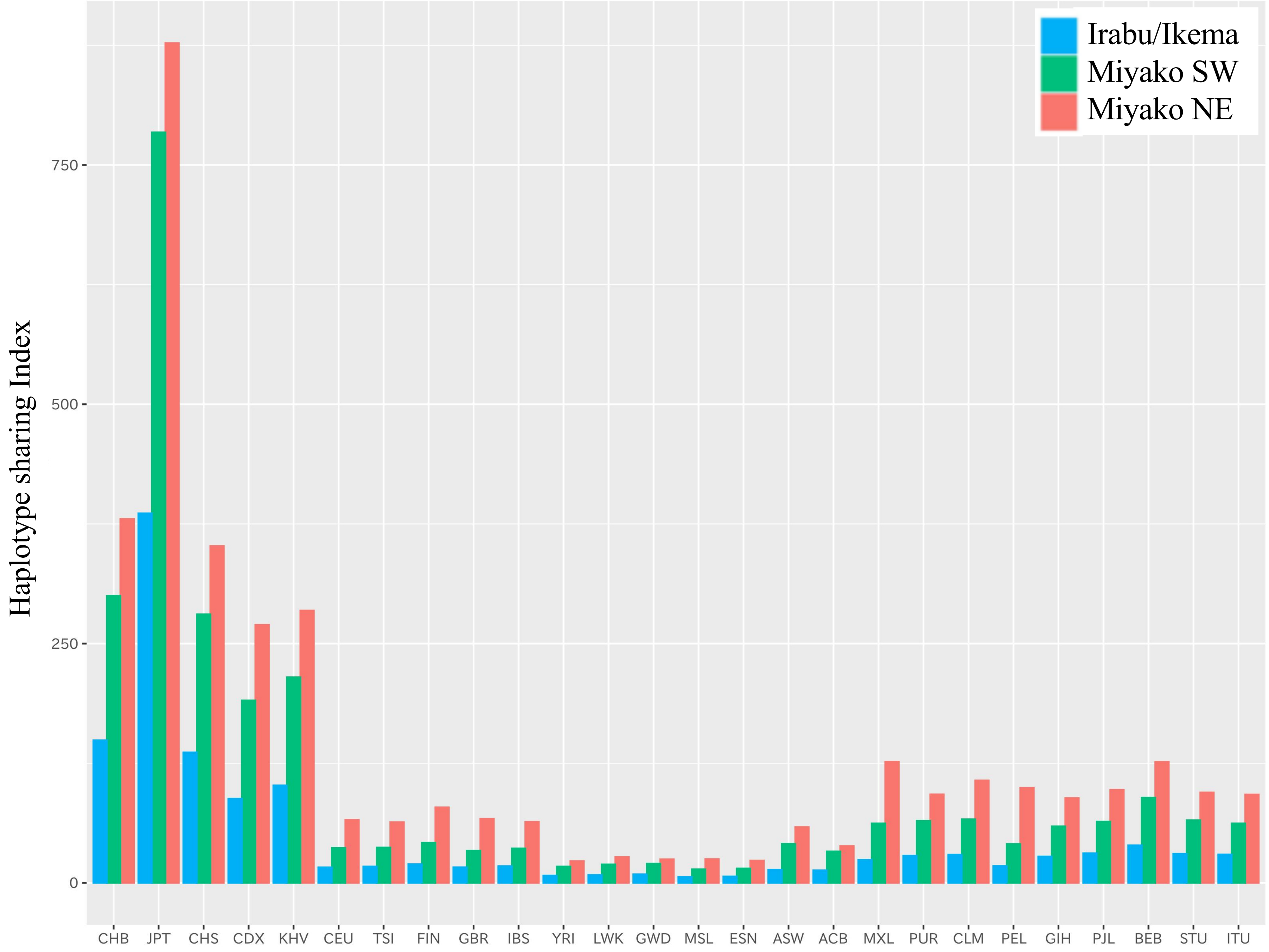

Supplementary Figure 8

Supplementary Figure 8. Haplotype sharing of each Miyako subpopulation with populations from the 1000 genomes project
We examined haplotype sharing between each Miyako subpopulation and populations from 1000 genomes project. Vertical axis represents sharing index between each pair of the populations. Blue, green, and red bars indicate the Irabu/Ikema, Miyakojima southwest, and Miyakojima northeast subpopulations, respectively. Horizontal axis shows each population from the 1000 genomes project. CHB: Han Chinese in Beijing, China, JPT: Japanese in Tokyo, Japan, CHS: Southern Han Chinese, CDX: Chinese Dai in Xishuangbanna, China, KHV: Kinh in Ho Chi Minh City, Vietnam, CEU: Utah Residents with Northern and Western European Ancestry, TSI: Toscani in Italia, FIN: Finnish in Finland, GBR: British in England and Scotland, IBS: Iberian Population in Spain, YRI: Yoruba in Ibadan, Nigeria, LWK: Luhya in Webuye, Kenya, GWD: Gambian in Western Divisions in the Gambia, MSL: Mende in Sierra Leone, ESN: Esan in Nigeria, ASW: Americans of African Ancestry in SW USA, ACB: African Caribbeans in Barbados, MXL: Mexican Ancestry from Los Angeles USA, PUR: Puerto Ricans from Puerto Rico, CLM: Colombians from Medellin, Colombia, PEL: Peruvians from Lima, Peru, GIH: Gujarati Indian from Houston, Texas, PJL: Punjabi from Lahore, Pakistan, BEB: Bengali from Bangladesh, STU: Sri Lankan Tamil from the UK, ITU: Indian Telugu from the UK.

(A)

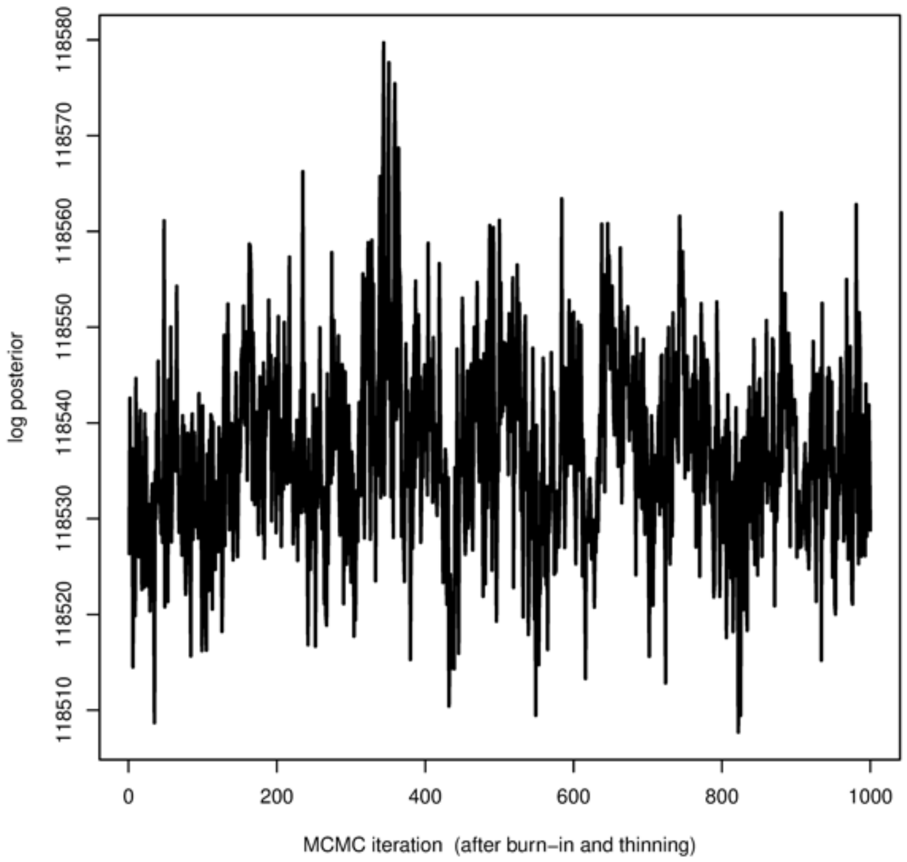

(B)

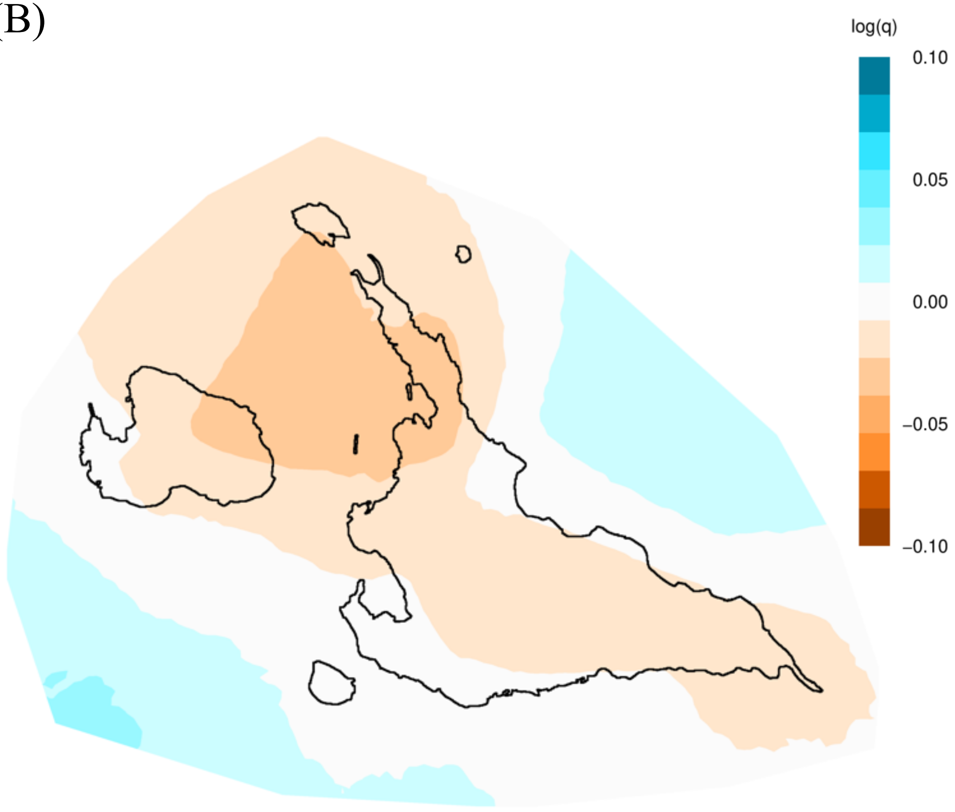

Supplementary Figure 9. EEMS output
(A) Posterior traces of MCMC runs. We confirmed that MCMC runs converged. (B) Estimated effective diversity surface plotted on the common logarithmic scale.

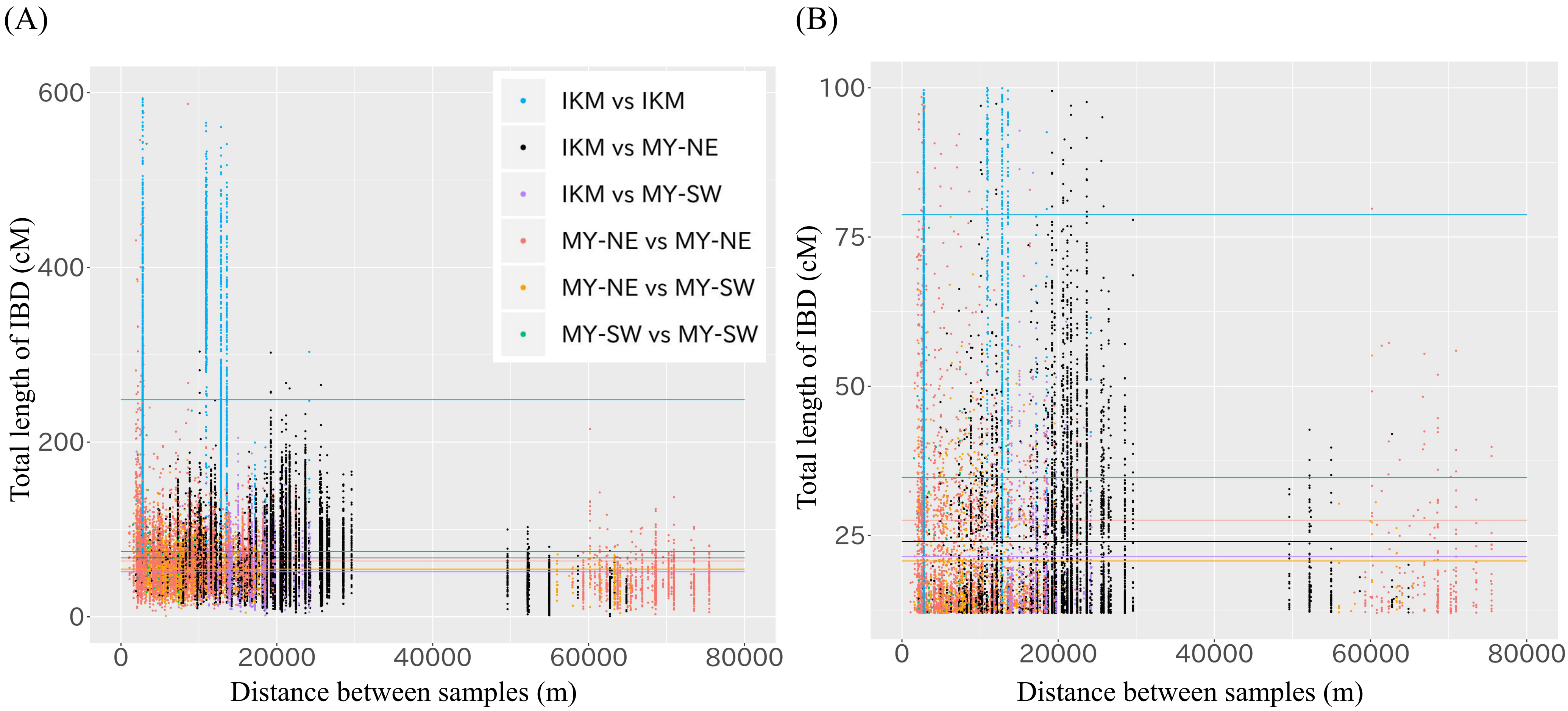

Supplementary Figure 10. Isolation by distance

We plotted total length of IBD for each pair and geographic distance. (A) All and (B) more than 12 cM IBD segments were used for calculation of total length. Horizontal axis represents geographic distance between each settlement, and vertical axis represents total length of shared IBD. Each horizontal line is mean total length of IBD for each pair.

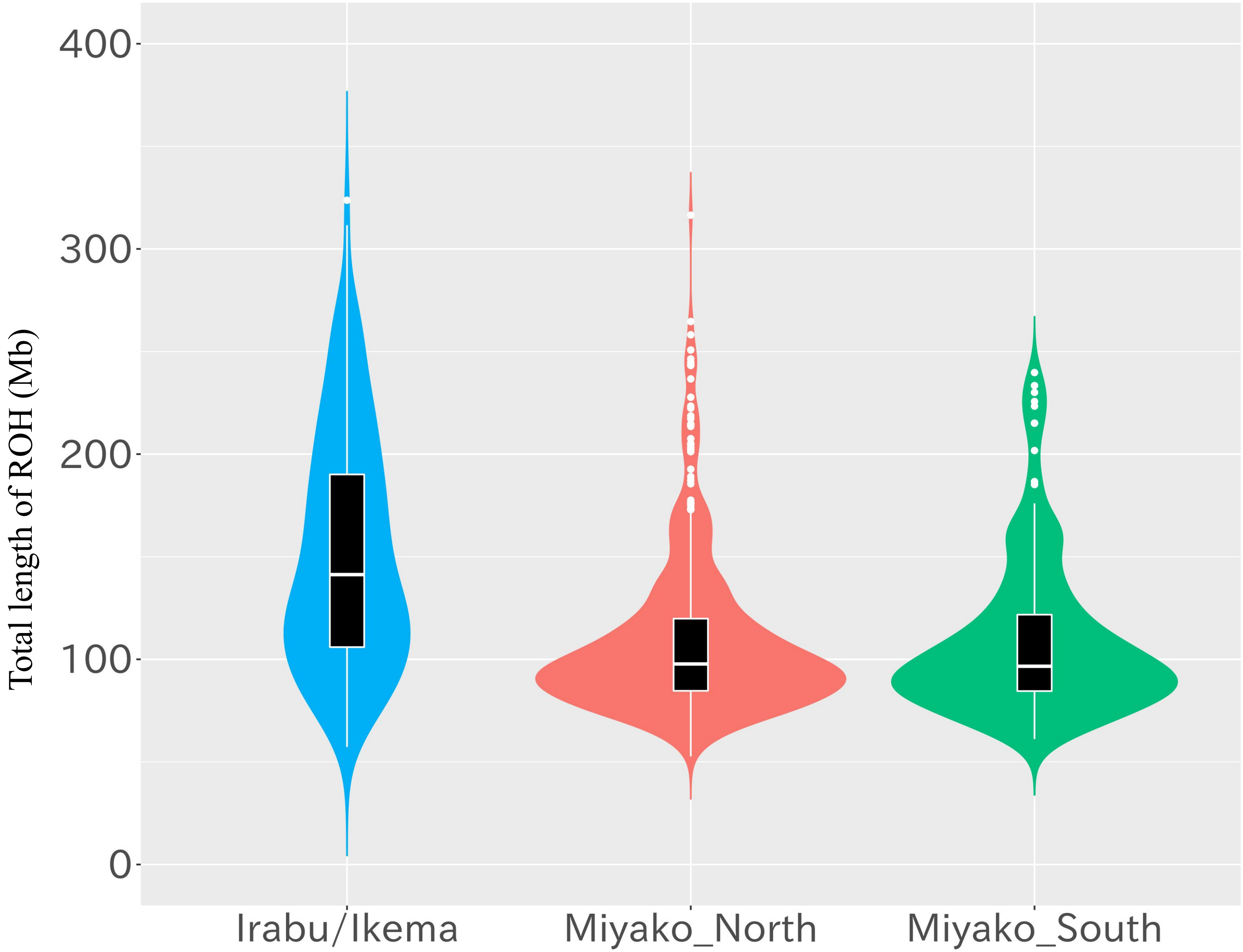

Supplementary Figure 11. Violin plot shows distribution of total length of ROH for each subpopulation with rotated kernel probability density. Each black box indicates the interquartile range and each horizontal white line indicates median.

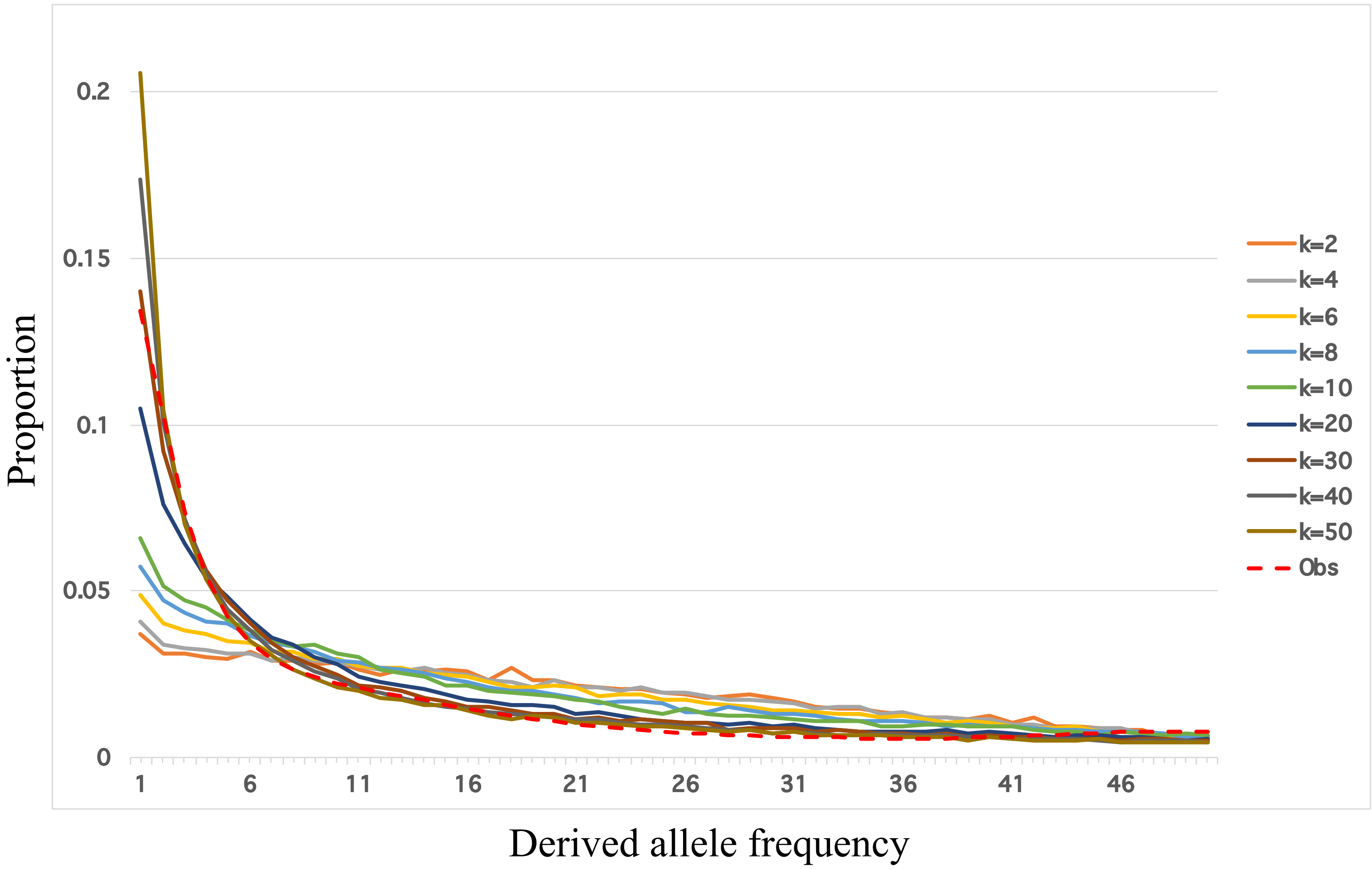

Supplementary Figure 12. Observed and expected unfold SFS using CHB population with different "discovery sample" size (*k*)
We inferred ascertainment bias of the Asian Screening Array before performing a coalescent simulation using the CHB population. We plotted observed and expected SFS under several "discovery sample" sizes (k= 2, 4, 6, 8, 10, 20, 30, 40, and 50) using fastsimcoal2 ver 2.6.0.3 program with 10,000 coalescent iterations.

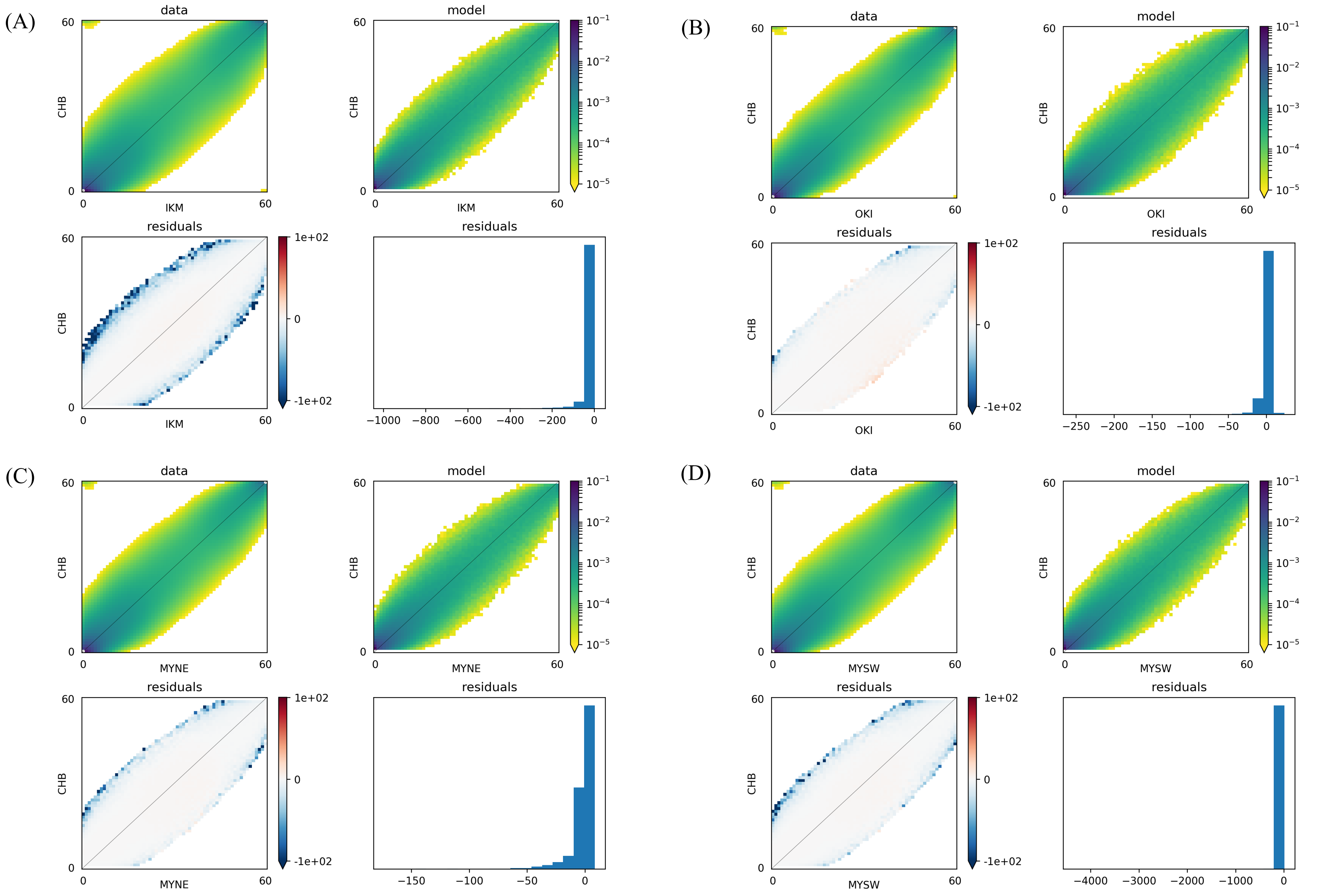

Supplementary Figure 13. Observed and expected joint site frequency spectra (SFS) between the CHB and the Miyako subpopulations
Based on fastsimcoal2 analysis, observed and excepted joint site frequency spectra of each population pair and the Anscombe residuals between each population pair were calculated. We plot frequency of each bin (range: 1e-05 – 1e-01).

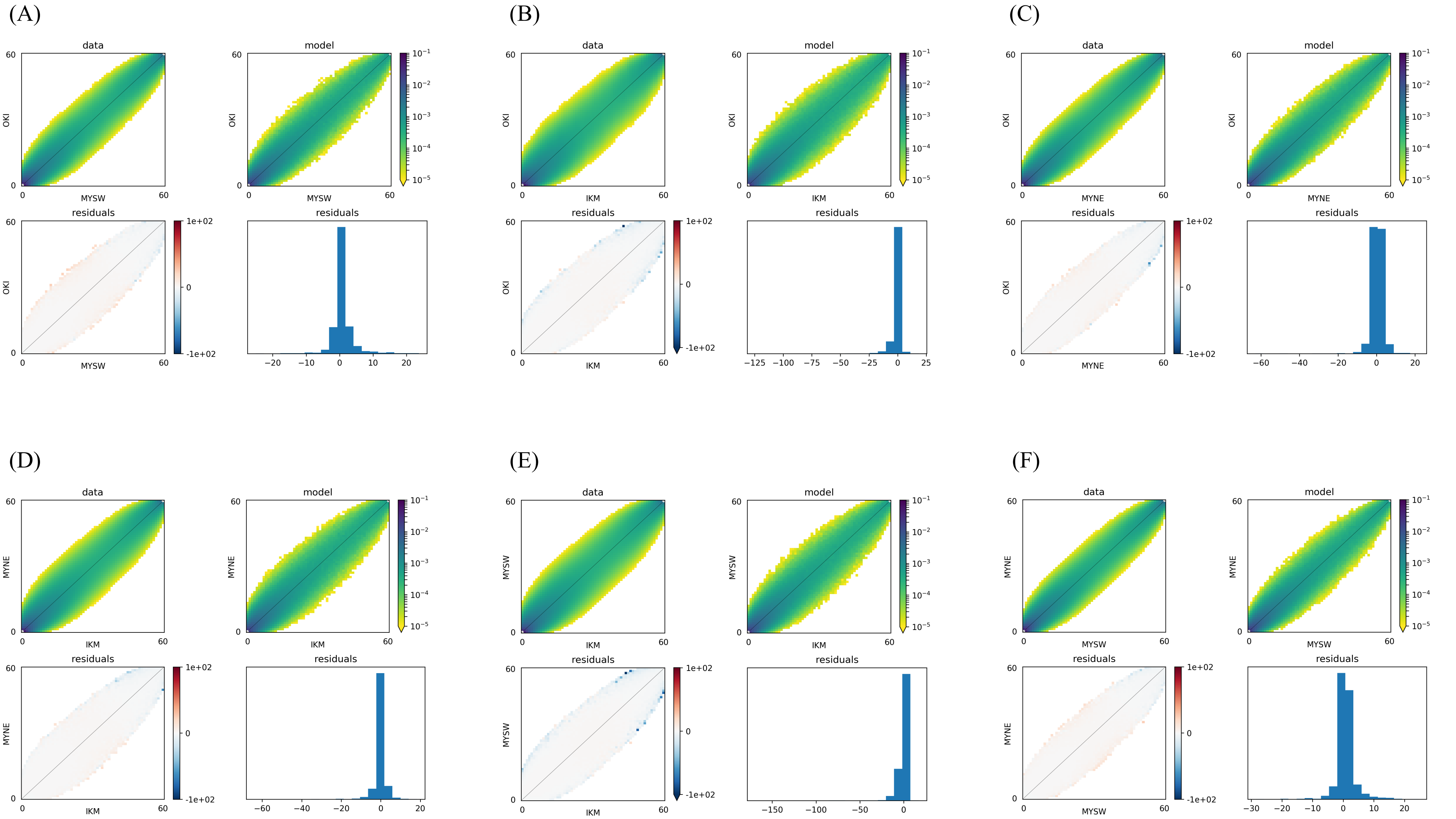

Supplementary Figure 14. Observed and expected joint SFS between the Miyako subpopulations

Based on fastsimcoal2 analysis, observed and excepted joint site frequency spectra of each population pair and the Anscombe residuals between each population pair were calculated. We plot frequency of each bin (range: 1e-05 – 1e-01).
