# Supplementary material for: Fine-Scale Genetic Structure and Demographic History in the Miyako Islands of the Ryukyu Archipelago
Source: Mol Biol Evol. 2021 Jan 12;38(5):2045–56. doi: 10.1093/molbev/msab005 (PMC8097307; doi:10.1093/molbev/msab005)
Supplement: msab005_Supplementary_Data [file msab005_supplementary_data.zip › Miyako_SI.pdf]

Supplementary Information

A. ADMIXTURE analysis

To estimate individual ancestry, we subjected the 834 individuals to maximum-likelihood clustering analysis using ADMIXTURE (Alexander et al. 2009). We ran ADMIXTURE from K = 2 to 8 and calculated the cross-validation errors for each value of K.

In this analysis, cross-validation error for K = 2 is smallest, implying that the model of two subpopulations is most suitable for explaining the population structure within the Miyako Islands (Supplementary Fig. 1). The components of these two subpopulations were mainly individuals from Miyakojima and Irabu/Ikema, respectively. However, the difference in cross-validation error between K = 2 and K = 3 is very small, and we cannot exclude the possibility that a three-subpopulation model is more suitable for explaining the population structure within the Miyako Islands.

B. Isolation by distance

We plotted shared identity by descent (IBD) segments against geographic distance to infer isolation by distance within Miyako Islands. The length of shared IBD segments between each locality was calculated using IBD-seq and converted physical length (in base pairs) to genetic distance (in cM) (see Haplotype sharing section). Distances between local populations were calculated based on geographic information (longitude and latitude).

We plotted total length of IBD for each pair according to the geographic distance (Supplementary Fig. 10). All and more than 12 cM IBD segments were used for calculation of total length. When we picked up longer IBD segments, signals among

subpopulations could become clearer. We found contribution of the geographic distance to genetic differentiation in the Miyako populations is very weak using all IBD segments (adjusted R-square = 0.067; $p < 2.2e^{-16}$), and total length of IBD among individual pairs showed similar pattern regardless of the geographic distance. Among pairs examined, we observed higher total length of IBD among Irabu/Ikema subpopulation (mean = 248.59 cM), suggesting relatively high inbreeding in this subpopulation.

C. Methods for Inferring the population genetic structure within Miyako Islands

To infer a more detailed population structure in the Miyako Islands, 834 individuals having all four grandparents born in the Miyako Islands were included for further analyses. We carried out PCA using these 834 individuals and confirmed that there are no population outliers among the 834 individuals (PC1 and PC2 values of all samples are within mean ± 6 s.d.).

To infer the population structure in greater detail, we used the haplotype-based clustering method in the FineSTRUCTURE/ChromoPainter program (v4.0.1) in which individuals were assigned into genetically homogenous groups using a nonparametric Bayesian mixture model implemented through a MCMC algorithm (Lawson et al. 2012). We prepared phased genotype data using EAGLE v2.4.1 (Loh et al. 2016). We ran the FineSTRUCTURE MCMC model for burn-in of 2,000,000 and 2,000,000 sampling iterations from which every 100,000th iteration was recorded. We then ran an additional 100,000 hill-climbing steps before conducting tree reconstruction to improve posterior probability. After defining subpopulations in the Miyako Islands according to FineSTRUCTURE clustering, the Hudson's F_{ST} values (Hudson et al. 1992; Bhatia et al.

2013) between individuals from Miyako subpopulations, Okinawajima, and Hondo, were calculated using the KRIS software package (version 1.1.1, R version 3.6.2; https://rdrr.io/cran/KRIS/). We used modified function "fst.hudson" for calculation of F_{ST} with standard error. In addition, we calculated total length of ROH in each individual using PLINK 1.9 program with default setting.

D. Methods for Demographic inferences by fastsimcoal2

We chose four populations (Okinawajima, Miyakojima northeast, Miyakojima southwest, and Irabu/Ikema) along with CHB from the 1000 genomes project as an outgroup for the coalescent simulation. To obtain SNPs reflecting historical demographic events, we focused on neutral biallelic SNPs. We discarded SNPs located in the protein-coding regions based on the annotation by the ANNOVAR program (Wang et al. 2010). We also discarded SNPs in CpG islands according to the UCSC platform (Rosenbloom et al. 2015). After filtering, the ancestral states of each SNP were inferred using the ancestral hg19 genome provided by the 1000 genomes consortium (The 1000 Genomes Project Consortium 2012). This pipeline estimated the ancestral state of the human genome using the alignment of six primate species based on Ensembl Compara 59 database (Flicek et al. 2011). We retained 969 individuals with 290,815 SNPs with no missing data. This data was converted into unfold site frequency spectra (SFS) by the script "easySFS.py" (https://github.com/isaacovercast/easySFS). To avoid sampling bias, we randomly chose 60 haplotypes for each population using the projecting populations parameter of this script.

Since the ascertainment bias of the SNP array distorts SFS (Marth et al. 2004), we need to correct the bias by using the "discovery sample" size. However, the actual

"discovery sample" size for the Asian Screening Array is unknown. Therefore, we preliminarily inferred the "discovery sample" size for the Asian Screening Array using the CHB population. We assumed ancient bottleneck events due to out-of-Africa and recent population growth for the CHB population. Assuming a fixed demographic model for CHB, we simulated SFS under several k values (*k*= 2, 4, 6, 8, 10, 20, 30, 40, and 50) using fastsimcoal2 ver 2.6.0.3 program (Excoffier et al. 2013), and compared them with the observed SFS by the least-square approach. Then, we found that k = 30 was most suitable to correct the ascertainment bias of the SNP array (Supplementary Fig. 12).

We inferred the demographic population history, namely effective population sizes, migration rates and divergence times under the assumption of recent migrations among the Ryukyu populations. We proposed a demographic model considering the population size changes inferred from our haplotype-based analysis (Fig. 8). The parameters related to the outgroup population (CHB) were fixed. Other parameters related to the Ryukyu populations, including effective population size, time of each event, and migration rates, were estimated by demographic inferences. Input parameter files for fastsimcoal2 analysis were described in the end of this Supplementary Information. The joint unfold SFS of each population pair was prepared by the script "easySFS.py". These were utilized for coalescent simulation using fastsimcoal2 ver 2.6.0.3 program. One hundred independent fastsimcoal2 runs with broad prior search ranges for each parameter were performed. Each run was comprised 40 rounds of parameter estimation via the expectation/conditional maximization algorithm with a length of 100,000 coalescent simulations each. Based on the likelihood values of each run, the best-fit model was selected. Using dadi program (Gutenkunst et al. 2009), we plotted each observed and expected joint SFS and the Anscombe residuals between each population pair

(Supplementary Figs 13-14). A mutation rate of 1.25e-8 per generation and per site were assumed (Scally and Durbin 2012).

Then, we estimated the 95 % percentile CI of each parameter using a nonparametric block-bootstrap approach (Meier et al. 2016). Considering the linkage disequilibrium, we divided our SNP data into 100 blocks, and prepared bootstrap data by randomly sampled SNPs from each block. We took 50 block-bootstrapping replicates, and we estimated the 95 % percentile CI from the bootstrap data.

References

Alexander DH, Novembre J, Lange K. 2009. Fast model-based estimation of ancestry in unrelated individuals. *Genome Res.* 19:1655–64.

Bhatia G, Patterson N, Sankararaman S, Price AL. 2013. Estimating and interpreting F_{ST}: the impact of rare variants. *Genome Res.* 23:1514-21.

Excoffier L, Dupanloup I, Huerta-Sánchez E, Sousa VC, Foll M. 2013. Robust demographic inference from genomic and SNP data. *PLoS Genet.* 9:e1003905.

Flicek P, Amode MR, Barrell D, Beal K, Brent S, Carvalho-Silva D, Clapham P, Coates G, Fairley S, Fitzgerald S, et al. 2012. Ensembl 2012. *Nucleic Acids Res.* 40(Database issue):D84-90.

Gutenkunst RN, Hernandez RD, Williamson SH, Bustamante CD. 2009. Inferring the joint demographic history of multiple populations from multidimensional SNP frequency data. *PLoS Genet.* 5:e1000695.

Hudson RR, Slatkin M, Maddison WP. 1992. Estimation of levels of gene flow from DNA sequence data. *Genetics.* 132:583-9.

Lawson DJ, Hellenthal G, Myers S, Falush D. 2012. Inference of population structure using dense haplotype data. *PLoS Genet.* 8:e1002453.

Loh PR, Danecek P, Palamara PF, Fuchsberger C, A Reshef Y, K Finucane H, Schoenherr S, Forer L, McCarthy S, Abecasis GR, et al. 2016. Reference-based phasing using the Haplotype Reference Consortium panel. *Nat Genet.* 48:1443-1448.

Marth GT, Czabarka E, Murvai J, Sherry ST. 2004. The allele frequency spectrum in genome-wide human variation data reveals signals of differential demographic history in three large world populations. *Genetics.* 166:351-72.

Meier JI, Sousa VC, Marques DA, Selz OM, Wagner CE, Excoffier L, Seehausen O. 2017 Demographic modelling with whole-genome data reveals parallel origin of similar *Pundamilia* cichlid species after hybridization. *Mol Ecol.* 26:123-141.

Rosenbloom KR, Armstrong J, Barber GP, Casper J, Clawson H, Diekhans M, Dreszer TR, Fujita PA, Guruvadoo L, Haeussler M, et al. 2015. The UCSC Genome Browser database: 2015 update. *Nucleic Acids Res.* 43(Database issue):D670-81.

Scally A, Durbin R. 2012. Revising the human mutation rate: implications for understanding human evolution. *Nat Rev Genet.* 13:745-53.

Wang K, Li M, Hakonarson H. 2010. ANNOVAR: functional annotation of genetic variants from high-throughput sequencing data. *Nucleic Acids Res.* 38:e164.

Template (.tpl) file for fastsimcoal2

```
//Parameters for the coalescence simulation program
5
//Population effective sizes (number of genes)
200000 //CHB
NOKI1    //OKI
NMYNE    //MYNE
NMYSW    //MYSW
NIKM1    //IKM
//Samples sizes and samples age
60
60
60
60
60
//Growth rates: negative growth implies population expansion
0
OKIR1
0
0
IKMR1
//Number of migration matrices : 0 implies no migration between demes
2
```

```
//Migration matrix 0
0 0 0 0 0
0 0 MOKIMYNE MOKIMYSW MOKIIKM
0 MOKIMYNE 0 MMYNEMYSW MMYNEIKM
0 MOKIMYSW MMYNEMYSW 0 MMYSWIKM
0 MOKIIKM MMYNEIKM MMYSWIKM 0
//Migration matrix 1
0 0 0 0 0
0 0 0 0 0
0 0 0 0 0
0 0 0 0 0
0 0 0 0 0
//historical event: time, source, sink, migrants, new deme size, growth rate, migr mat index
10 historical event
TRESIKM 4 4 0 1 0 1                    //Set growth rate to 0 in IKM
TRESIKM 4 4 0 IKMRES 0 1          //IKM resize
TDIVMYSW 3 2 1 1 0 1                  //MYSW split from RYU
TDIVIKM 4 2 1 1 0 1                    //IKM split from RYU
100 0 0 0 0.1 0 1                         //CHB resize
TDIVOKI 2 2 0 MYRES 0 1            //MYNE resize
TDIVOKI 1 2 1 1 0 1                    //OKI split from RYU
TDIVRYU 2 0 1 1 0 1                    //RYU split from CHB
2000 0 0 0 0.2 0 1                       //CHB expansion
```

```
2100 0 0 0 5 0 1                    //CHB bottleneck
//Number of independent loci [chromosome]
1 0
//Per chromosome: Number of contiguous linkage Block: a block is a set of contiguous loci
1
//per Block:data type, number of loci, per gen recomb and mut rates
FREQ 1 0 1.25e-8 OUTEXP
```

Distribution (.est) file for fastsimcoal2

```
// Priors and rules file
// *********************

[PARAMETERS]
//#isInt? #name	#dist.#min	#max
//all Ns are in number of haploid individuals
1	NOKI1	unif	20000	40000	output
1	NOKI2	unif	2000	20000	output
1	NMYNE	unif	20000	40000	output
1	NRYU	unif	2000	20000	output
1	NMYSW	unif	2000	20000	output
1	NIKM1	unif	2000	20000	output
1	NIKM2	unif	2000	20000	output
1	NIKM3	unif	2000	20000	output
1	TANCEST	unif	100	1000	hide
1	TDIVMYSW	unif	1	20	output
1	TMYMID	unif	1	20	hide
1	TRESIKM	unif	1	20	output
1	TIKMMID	unif	1	20	hide
1	TDIVOKI	unif	1	100	output
0	MMYNEMYSW	logunif	1e-10	1e-1	output
0	MMYNEIKM	logunif	1e-10	1e-1	output
```

```
0    MMYSWIKM    logunif    1e-10 1e-1      output
0    MOKIMYNE    logunif    1e-10 1e-1      output
0    MOKIMYSW    logunif    1e-10 1e-1      output
0    MOKIIKM     logunif    1e-10 1e-1      output

[RULES]


[COMPLEX PARAMETERS]
1    TDIVRYU         = TDIVOKI+TANCEST         output
1    TDIVIKM         = TDIVMYSW+TMYMID         output
0    OKIRATIO_EXP    = NOKI2/NOKI1             hide
0    OKIRTEA         = log(OKIRATIO_EXP)       hide
0    OKIR1           = OKIRTEA/TDIVOKI         hide
0    MYRES           = NRYU/NMYNE              hide
0    IKMRES          = NIKM3/NIKM2             hide
0    IKMRATIO_BOT    = NIKM2/NIKM1             hide
0    IKMRTEA         = log(IKMRATIO_BOT)       hide
0    IKMR1           = IKMRTEA/TRESIKM         hide
```
